# An Organometallic Erbium Bismuth Cluster Complex Comprising a Bi_6_^6–^ Zintl Ion

**DOI:** 10.1021/acs.inorgchem.4c02636

**Published:** 2024-10-18

**Authors:** Florian Benner, Elizabeth R. Pugliese, Reece Q. Marsden, Richard J. Staples, Nicholas F. Chilton, Selvan Demir

**Affiliations:** † Department of Chemistry, Michigan State University, 578 South Shaw Lane, East Lansing, Michigan 48824, United States; ‡ Research School of Chemistry, The Australian National University, Sullivans Creek Road, Canberra, ACT 2601, Australia; § Department of Chemistry, The University of Manchester, Manchester M13 9PL, U.K.

## Abstract

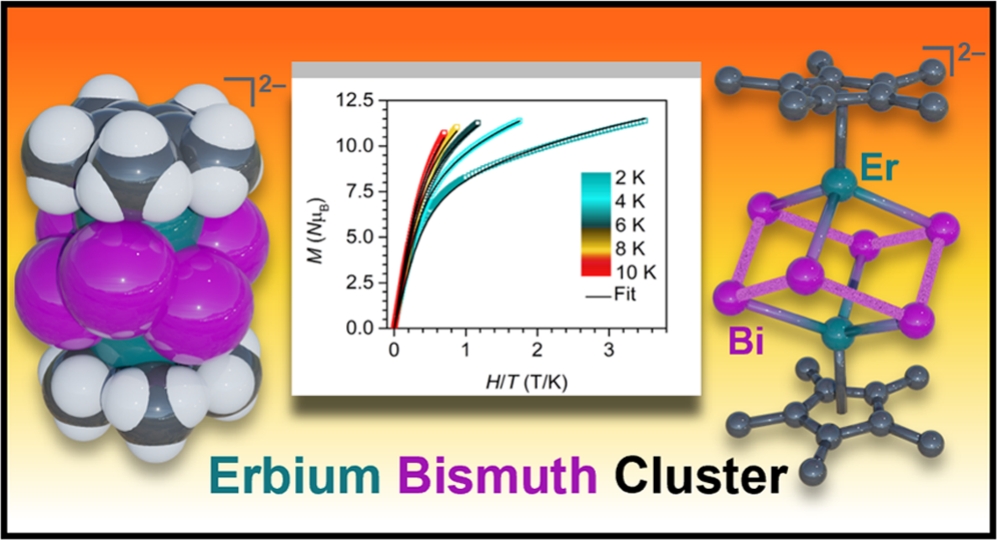

An organometallic erbium bismuth cluster complex, [K(THF)_4_]_2_[Cp*_2_Er_2_Bi_6_] (**1**), featuring a heterometallocubane core was isolated. The cube emerges from the rare Bi_6_^6–^ Zintl ion, bridging two erbium centers for the first time. SQUID magnetometry and *ab initio* calculations uncovered dominant antiferromagnetic coupling enabled through the chair-like hexabismuth anion. The gained insight will promote the design of future polynuclear magnetic molecules comprising prolate lanthanide ions.

## Abstract 2

Strong magnetic exchange coupling is observed in an unprecedented organometallic erbium bismuth cluster complex. Magnetometry and *ab initio* calculations revealed a favorable equatorial ligand field for the prolate Er^III^ ion, providing advanced design strategies to erbium-based magnetic materials including bismuth donors.

## Introduction

Zintl phases are intermetallic solid-state compounds comprising of two entities: (a) electropositive alkali/earth alkali/rare earth (RE) metals and (b) well-defined, polyanionic moieties of electronegative p-block elements. This fascinating class of inorganic compounds attract by virtue of unique electronic structures, rendering them intriguing for various applications such as semiconductors and thermoelectric and magnetic materials.^1,2^ Methods for accessing intermetallic clusters containing Zintl ions from reactions of multimetallic Zintl ion salts such as [K(crypt-222)]_2_Bi_2_ or K_5_Ga_2_Bi_4_ with d- or f-block metal compounds [CpRu(MeCN)_3_][PF_6_],^3^ or [Cp^tet^_3_ThCl] (Cp^tet^ = C_5_Me_4_H),^4^ ushered in a wealth of polymetallic cluster geometries with distinct properties, relevant for a broad range of applications such as white-light emission or single-molecule magnetism.^5−9^

The traditional synthesis of Zintl clusters treats metal precursor complexes with well-defined Zintl ion salts which are obtained through extraction from their solid-state phases such as the extraction of [K(L)]_2_Bi_2_ (where L = 2.2.2-cryptand or 18-crown-6) from K_5_Bi_4_.^10,11^ This latter extraction process typically involves polar protic solvents such as ethylenediamine, rendering it harsh and incompatible with most organometallic compounds. Recently, we developed a synthetic method to generate organometallic cluster complexes containing bismuth Zintl ions that are soluble in polar, aprotic solvents such as THF.^7,12^ The synthesis of these polybismuth cluster molecules [K(THF)_4_]_2_[Cp*_2_RE_2_Bi_6_] (where RE = Tb, Dy, Y; Cp* = pentamethylcyclopentadienyl)^7,12^ proceeds through excision of bismuth ions from organic compounds in the presence of rare earth ions and strongly reducing alkali metal reagents.^7,12,13^ Specifically, following chemical reduction, a homolytic cleavage of all three bismuth–carbon bonds in triphenylbismuth assembled a Bi_6_^6–^ Zintl ion which is bridging two RE ions forming a rare heterometallocubane, sandwiched between two Cp* rings, composing the anionic complex part [Cp*_2_RE_2_Bi_6_]^2–^. This solution-phase methodology to construct polybismuth compounds can be generalized, and we were subsequently able to isolate Bi_2_^2–^- and Bi_2_^3–•^-containing complexes (Cp*_2_RE)_2_(μ-η^2^:η^2^-Bi_2_) and [K(crypt-222)][(Cp*_2_RE)_2_(μ-η^2^:η^2^-Bi_2_)] (where RE = Gd, Tb, Dy, Y).^13^ Notably, the diamagnetic Bi_2_^2–^ and Bi_6_^6–^ bridges promote relatively strong superexchange between paramagnetic lanthanide ions, enabled through the diffuse valence 6p orbitals of bismuth, that altogether may constitute a promising design principle for higher nuclearity molecule-based magnetic materials.

**Scheme 1:**
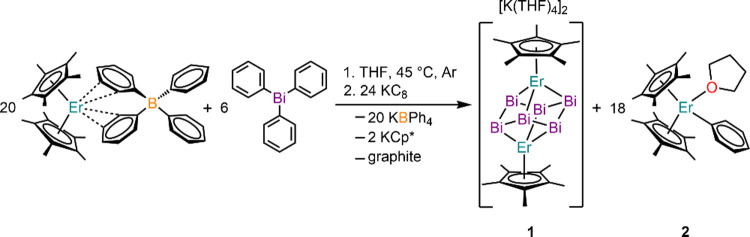
Synthetic Route for **1** through the Reduction of Trivalent BiPh_3_ to Bi^I–^

Interestingly, [K(THF)_4_]_2_[Cp*_2_RE_2_Bi_6_] (RE = Tb, Dy) are single-molecule magnets (SMMs) despite the equatorial nature of the Bi_6_^6–^ Zintl ion competing with the axial Cp* ligands, as the Bi_6_^6–^ cluster promotes ferromagnetic superexchange despite the predicted antiferromagnetic dipolar coupling arising from the single-ion magnetic anisotropy of the Dy^III^ and Tb^III^ ions.^7^ Accordingly, the Bi_6_^6–^ Zintl ion should provide more favorable magnetic anisotropy with a lanthanide ion with the opposite inherent sense of magnetic anisotropy. For Er^III^, the crystal field splitting of the *J* = ^15^/_2_ ground multiplet can be maximized in the presence of a strong equatorial ligand field, which stabilizes the prolate *m*_*J*_ = ±^15^/_2_ states while destabilizing the oblate *m*_*J*_ = ±^1^/_2_ states.^14−16^ Hence, we set out to probe the Er^III^ analogue to explore the interplay of single-ion anisotropy and magnetic exchange coupling. From a purely synthetic consideration, the ionic radii and RE^III^/RE^II^ reduction potentials (compare −3.1 V Er^III^/Er^II^ vs −2.8 V Y^III^/Y^II^) are similar,^17^ allowing one to presume an akin reaction progression when subjected to analogous conditions. Here, we present the isolation and characterization of [K(THF)_4_]_2_[Cp*_2_Er_2_Bi_6_] (**1**), which was synthesized from the reaction of Cp*_2_Er(BPh_4_) with triphenylbismuth at 45 °C and the reducing agent potassium graphite (KC_8_; Scheme 1). The latter formally reduces Bi^III^ to Bi^I–^ species affording two {Cp*Er}^2+^ cations and a Bi_6_^6–^ Zintl ion which combine into a dianionic complex with solvated [K(THF)_4_]^+^ cations in the outer sphere (Figure S1). Complex **1** is solely THF-soluble facilitating the separation from the hexane-soluble byproduct Cp*_2_ErPh(THF) (**2**; Figure S3) and insoluble KBPh_4_ and graphite. Diffusion of diethyl ether into a concentrated THF solution at −35 °C yielded **1** as black block-shaped crystals.

## Experimental Section

### General Information

The manipulations described herein were performed under an inert argon atmosphere with rigorous exclusion of air and water using Schlenk line and glovebox techniques. *n*-Hexane was dried over calcium hydride. Diethyl ether and THF were dried over a Na/K alloy. Toluene was dried over potassium. All solvents were subsequently distilled, and transferred to an argon-filled glovebox. A drop of sodium benzophenone radical solution was added as an indicator to a given solvent to prove the absence of water and oxygen within the glovebox. Potassium bis(trimethylsilylamide) (K[N(SiMe_3_)_2_]) was purchased from Sigma-Aldrich, dissolved in toluene, centrifuged, filtered, and recrystallized at −35 °C. Triphenylbismuth (BiPh_3_) was purchased from Sigma-Aldrich and recrystallized from hexane at −35 °C. 1,2,3,4,5-Pentamethylcyclopentadiene (HCp*) was purchased from Sigma-Aldrich and dried over 4 Å sieves. Anhydrous erbium chloride (ErCl_3_) and allylmagnesium chloride (2.0 M in THF) were purchased from Sigma-Aldrich and used as received. KCp* was synthesized by deprotonation of HCp* with K[N(SiMe_3_)_2_].^18^ KC_8_,^19^ (HNEt_3_)(BPh_4_),^20^ and Cp*_2_Er(BPh_4_)^21^ were prepared according to literature procedures. CHN analysis was performed at Michigan State University using a PerkinElmer 2400 Series II CHNS/O analyzer.

### [K(THF)_4_]_2_[Cp*_2_Er_2_Bi_6_] (**1**)

In an argon-filled glovebox, Cp*_2_Er(BPh_4_) (100.6 mg, 0.1329 mmol) was dissolved in 2 mL of THF, forming a pale-pink solution, followed by the addition of a colorless solution of BiPh_3_ (17.5 mg, 0.0397 mmol) in 2 mL of THF. Subsequently, KC_8_ (21.7 mg, 0.160 mmol) was added at once to the reaction mixture and stirred for 15 min at 45 °C. The dark-red solution was filtered to remove the black and colorless insoluble solids, presumably graphite and KBPh_4_. The solvent was removed under vacuum to afford a dark solid, which was washed twice with 1 mL of hexane and twice with 2 mL of toluene to remove the byproduct Cp*_2_ErPh(THF) (**2**). The remaining black solid was dissolved in THF and filtered to obtain a dark-brown solution, which was subsequently evaporated to dryness. The solids were dissolved in 1 mL of THF. Black, block-shaped crystals of **1**, suitable for single-crystal X-ray diffraction (XRD) analysis were crystallized from the THF solution via vapor diffusion of Et_2_O at −35 °C in 19% yield, based on BiPh_3_. Anal. Calcd for Er_2_Bi_6_C_20_H_30_K_2_·THF: C, 14.35; H, 1.91; N, 0.0. Found. C, 14.13; H, 1.98; N, 0.06. Anal. Calcd for ErC_30_H_43_O: C, 61.39; H, 7.38; N, 0.0. Found. C, 61.02; H, 7.57; N, 0.07. IR (ATR, cm^–1^): 2835 (br), 1423 (m), 1369 (s), 1150 (s), 1094 (s), 1020 (s), 951 (s), 891 (w), 841 (s), 799 (s), 743 (w), 713 (w).

### X-ray Crystallography

A black block-shaped crystal of **1**, 0.278 × 0.175 × 0.112 mm^3^, was mounted on a nylon loop with Paratone oil. Data were collected using a XtaLAB Synergy, Dualflex, HyPix diffractometer equipped with an Oxford Cryosystems low-temperature device, operating at *T* = 100.0(1) K. Data were measured using ω scans using Mo Kα radiation (microfocus sealed X-ray tube, 50 kV, 1 mA). The total number of runs and images was based on the strategy calculation from the program *CrysAlisPro* (Rigaku, V1.171.41.90a, 2020). Cell parameters were retrieved using *CrysAlisPro* (Rigaku, V1.171.41.90a, 2020) software and refined using *CrysAlisPro* (Rigaku, V1.171.41.90a, 2020). Data reduction was performed using the *CrysAlisPro* (Rigaku, V1.171.41.90a, 2020) software, which corrects for Lorentz polarization. A numerical absorption correction based on Gaussian integration over a multifaceted crystal model empirical absorption correction using spherical harmonics was implemented in the SCALE3 ABSPACK scaling algorithm.

The structure was solved in the space group *P*2_1_/*n* by using dual methods using the *ShelXT* (Sheldrick, 2015) structure solution program.^22^ The structure was refined by least-squares *ShelXL*(23) incorporated in the *OLEX2* software program.^24^ All Er and Bi atoms were refined anisotropically, but due to disorder and data quality refinement of the rest of the atoms were done isotropically. H atom positions were calculated geometrically and refined using the riding model.

More than three data sets of this compound have been collected on various crystallization attempts, with this being the best data to date. All data sets show a large cell and disorder providing for a lack of intensity of the data at high angle and the heavy atoms dominate the structure, evident by the second weight value in the weight statement. Refinement parameters are given in Table S1.

A yellow block-shaped crystal of **2**, 0.3 × 0.257 × 0.106 mm^3^, was mounted on a nylon loop with Paratone oil. Data were collected using a XtaLAB Synergy, Dualflex, HyPix diffractometer equipped with an Oxford Cryosystems low-temperature device, operating at *T* = 100.0(1) K. Data were measured using ω scans using Mo Kα radiation (microfocus sealed X-ray tube, 50 kV, 1 mA). The total number of runs and images was based on the strategy calculation from the program *CrysAlisPro* (Rigaku, V1.171.41.90a, 2020). Cell parameters were retrieved using *CrysAlisPro* (Rigaku, V1.171.41.90a, 2020) software and refined using *CrysAlisPro* (Rigaku, V1.171.41.90a, 2020). Data reduction was performed using the *CrysAlisPro* (Rigaku, V1.171.41.90a, 2020) software, which corrects for Lorentz polarization. A numerical absorption correction based on Gaussian integration over a multifaceted crystal model empirical absorption correction using spherical harmonics was implemented in the SCALE3 ABSPACK scaling algorithm.

The structure was solved in the space group *P*2_1_/*c* by using dual methods with the *ShelXT* (Sheldrick, 2015) structure solution program.^22^ The structure was refined by least-squares using version 20189/2 of *ShelXL*(23) incorporated in *OLEX2*.^24^ All non-H atoms were refined anisotropically. H atom positions were calculated geometrically and refined using the riding model.

Complexes of structure similar to that of **1** have been reported previously.^25^

### Powder XRD

XRD patterns were obtained on Bruker D8 DaVinci diffractometer equipped with Cu X-ray radiation operating at 40 kV and 40 mA. Peak intensities were obtained by counting with the Lynxeye detector every 0.02° at sweep rates of 1.4° 2θ /min (Table S2). The sample was placed on top of a zero-background sample holder. The sample was rotated at 5 degrees per minute. No background correction is applied to raw data. Using Bruker software that sample was matched to the corresponding Bi^0^ powder pattern.

### UV–Vis Spectroscopy

UV–vis spectra were collected with an Agilent Cary 60 spectrophotometer at ambient temperature from 200 to 1100 nm. Samples were prepared in an argon-filled glovebox and measured in a 1 cm quartz cuvette. The spectrum is baseline-corrected from a sample of dry THF.

### IR Spectroscopy

IR spectra were recorded with an Agilent Cary 630 ATR spectrometer under an inert nitrogen atmosphere.

### *Ab initio* Calculations

To probe the electronic structure of complex **1**, we employed state-average complete active space self-consistent-field spin–orbit (SA-CASSCF-SO) calculations in OpenMolcas 22.06.^26^ Unoptimized crystal structures of the [Cp*_2_Er_2_Bi_6_]^2–^ fragments obtained from single-crystal XRD experiments were used in calculations for each Er^III^ ion individually, where the Er^III^ ion out of scope was replaced with closed-shell Lu^III^. An active space of 11 4f electrons in 7 4f orbitals was considered including 35 quartets and 112 doublets, and all states were then mixed with spin–orbit coupling. The two electron integrals were truncated using a Cholesky decomposition of threshold 10^–8^ and relativistic effects were accounted for with the second order DKH Hamiltonian.^27,28^ ANO-RCC basis sets^29^ were used with VTZP quality for Er^III^, VDZP quality for Bi and bonded Cp* carbon atoms, and MB quality for all other atoms.

## Results and Discussion

**Figure 1:**
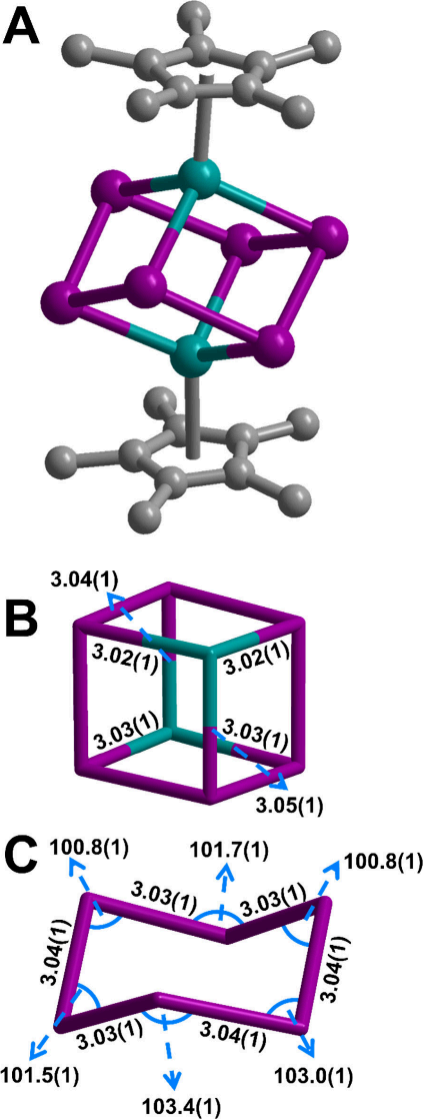
(A) Structure of the erbium bismuth cluster dianion in a crystal of **1**. Teal, purple, and gray spheres represent Er, Bi, and C atoms, respectively. H atoms and the [K(THF)_4_]^+^ cations have been omitted for clarity. (B) Neutral {Er_2_Bi_6_} heterometallocubane with Er–Bi distances labeled. (C) Bi_6_^6–^ chair with distances and angles labeled. Distances (Å) and angles (deg) are rounded up for clarity and full values can be found in the main text.

**Figure 2:**
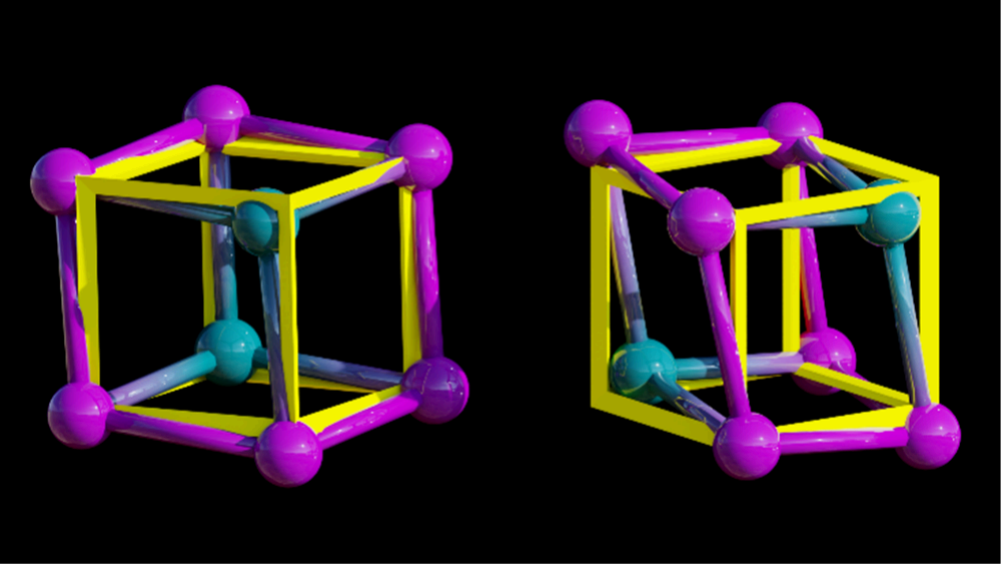
Superposition of an idealized cube (yellow) with a {Er_2_Bi_6_} ball-and-stick model in top (left) and side view (right), highlighting structural distortions in **1**.

Complex **1** crystallizes in the monoclinic *P*2_1_/*n* space group and features two Er^III^ ions, each capped by one Cp* ligand, and bridged by a Bi_6_^6–^ unit forming a distorted heterometallocubane (Figures 1 and S2). The Er–Bi distances in **1** of 3.022(1)–3.054(1) Å are slightly shorter than those of the corresponding Tb (3.06–3.07 Å), Dy (3.04–3.06 Å), and Y (3.04–3.20 Å) analogues, in line with the decreasing ionic radii traversing through the lanthanide series from Tb to Er.^7,12,30^ Relative to solid-state materials, the Er–Bi distances in **1** are considerably shorter, for instance, in comparison to the quaternary compound Bi_2_ErO_4_X, which shows Er–X distances of 3.684, 3.689, and 3.693 Å for X = Cl, Br, and I, respectively.^31,32^ The Bi_6_^6–^ moiety assumes a chair conformation, akin to cyclohexane, with Bi–Bi distances of 3.027(1)–3.041(1) Å, in line with Bi–Bi single bonds (>2.99 Å);^7,12,33^ Bi–Bi double bonds tend to be shorter, around 2.84 Å.^13,33,34^ The average Er–Cp_centroid_ distance of 2.331 Å is in accordance with that of other Er–Cp_centroid_ distances in half-metallocene cluster complexes, such as 2.349 Å as observed in [K(crypt-222)]_2_[(Cp*Er)_6_(μ_6_-κ^3^:κ^3^:κ^3^-Te_3_)(μ-κ^2^:κ^2^-Te_2_)(μ_3_-η^2^:κ:κ^1^-Te^2^)(μ_3_-Te)_3_] (crypt-222 = 4,7,13,16,21,24-hexaoxa-1,10-diazabicyclo[8.8.8]hexacosane).^100^ Furthermore, unlike the Y, Tb, and Dy analogues, **1** lacks an inversion center, giving rise to a more distorted heterometallocubane moiety. The Bi–Bi–Bi angles within the Bi_6_^6–^ unit in **1** vary by 3%, whereas in the Y and Dy analogues the angles differ by 2% and 1%, respectively (Table S3). Accordingly, with decreasing ionic radii of the RE metal, the cubane becomes more contorted traversing from Tb to Er.^7,12^ Consequently, the central cubane moiety in **1** differs from that of an idealized cube (Figures 2 and S4 and Table S3). In **1**, the angles at each corner range from a minimum of 76.3° (Er–Bi–Bi) to a maximum of 103.9° (Bi–Er–Bi), vastly deviating from that of a perfect cube with all angles equaling 90° (Figure S5).

UV–vis spectra of **1** were recorded at a concentration of 50 μmol L^–1^ in THF at room temperature over the course of 10 scans (Figures S7–S10). Each spectrum exhibits absorption over the entire range of the visible region. Similarly to the Y analogue, no absorption maxima are discernible.^12^ Very broad features are present in the visible region spanning from 607 to 445 nm in the first spectrum collected. However, after prolonged times, **1** decomposes in THF at room temperature prompting a disappearance of these features over the course of 10 scans. The decomposition of **1** can be attributed to the general instability of the complex in solution through the formation of black insoluble solids. To probe the nature of the decomposition product, powder XRD data were collected on the obtained insoluble solid, which coincides with the reported powder pattern of Bi^0^ (Figure S6).^35^ Such decomposition can be seen over prolonged periods of time at −35 °C in the absence of light.

**Figure 3:**
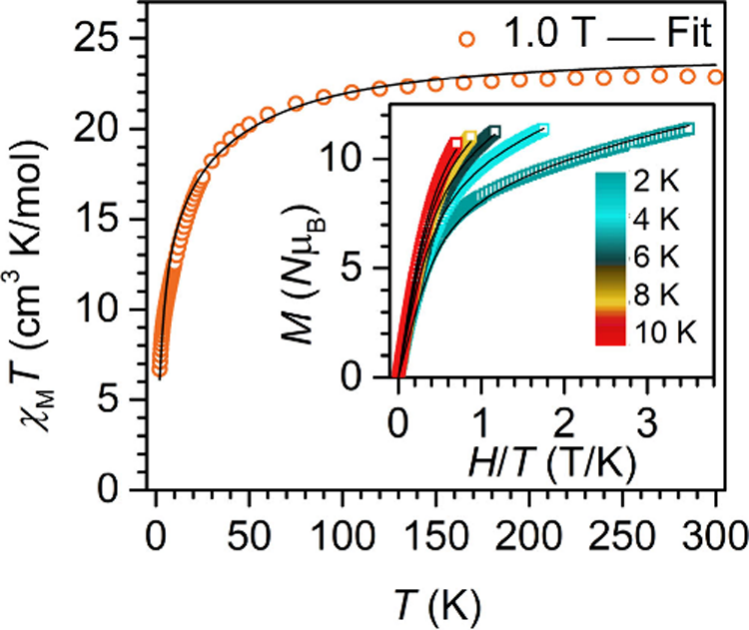
Variable-temperature dc magnetic susceptibility data for a restrained polycrystalline sample of **1**, collected under a 1.0 T applied dc field. Inset: Reduced magnetization data for **1**, collected between 0 and 7 T at 2, 4, 6, 8, and 10 K.

Direct current (dc) magnetic susceptibility data were collected on polycrystalline samples of **1** under applied magnetic fields of 0.5 and 1.0 T from 2 to 300 K (Figures 3 and S12 and S13). The room temperature χ_M_*T* value of 23.04 cm^3^ K mol^–1^ for **1** at 0.5 T (22.85 cm^3^ K mol^–1^ at 1.0 T) is in accordance with the expected value of 22.96 cm^3^ K mol^–1^ for two uncoupled Er^III^ ions (^4^*I*_15/2_ = 11.48 cm^3^ K mol^–1^). When the temperature is lowered, χ_M_*T* declines gradually until approximately 90 K, followed by a rapid decrease to 6.71 cm^3^ K mol^–1^, arising from depopulation of crystal field states and/or antiferromagnetic coupling. The isothermal field-dependent magnetization data (*M* vs *H*) were recorded at 2, 4, 6, 8, and 10 K up to 7 T. At each temperature, a steady increase in *M* is observed without reaching saturation, even at the highest fields (Figure S14). The maximum moment of 11.40 μ_B_ (2 K, 7 T) is significantly higher compared to other (di)erbium complexes, where values of ∼4.5–5.0 μ_B_ per Er^III^ ion are most common.^15,36−41^ Notably, the reduced magnetization (*M* vs *H*/*T*) plots exhibit exceptionally strong deviations from superposition, indicative of low-lying excited states that likely arise from exchange coupling between the two Er^III^ ions and/or crystal field splitting (Figures 3 and S17).

To investigate the relaxation dynamics of **1**, variable-frequency variable-temperature in-phase (χ_M_′) and out-of-phase (χ_M_″) alternating current (ac) magnetic susceptibility data were collected for a polycrystalline sample at zero and nonzero dc fields. No discernible peaks in χ_M_″ were observed at 1.8 K in any case, even with dc fields up to 5000 Oe. Hence, **1** lacks slow magnetic relaxation which is also reflected in an absence of magnetic hysteresis in variable-field magnetization experiments, where forward and backward scans are fully superimposable (Figure S15).

Moreover, the inner-cluster Er···Er distance in **1** is considerably shorter relative to iodide-bridged polynuclear Er complexes, where distances of 4.7873(7)/4.8264(7) in bis(η^2^-iodo)bis((η^8^-1,4-bis(triisopropylsilyl)cyclooctatetraenyl)tetrahydrofuranerbium) enabled a dipolar coupling of 0.65 cm^–1^.^16^ Within methyl bridged [ErCOT(Me)(THF)]_2_ (Er–Er: 3.5144(5) Å) and [(ErCOT)_2_(Me)_3_] (3.2533(8)/3.2450(7) Å) these couplings are of dipolar nature and augmented to 2.005 and 3.904 cm^–1^.^42^ Thus, a concurrence between exchange and dipolar coupling may be hypothesized to prevent the formation of pure exchange states in **1**.

**Figure 4:**
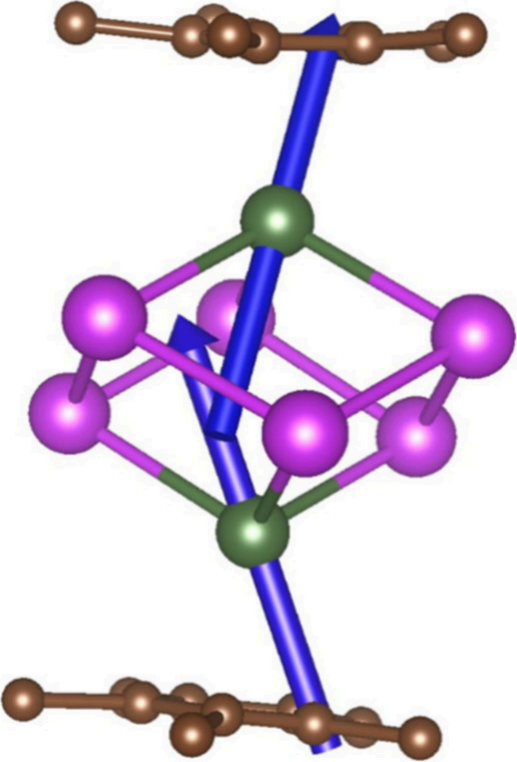
Orientations of the main easy axes of the ground doublets for Er^III^ ions from SA-CASSCF-SO calculations. Green, purple, and brown spheres represent Er, Bi, and C, respectively; H atoms omitted for clarity.

To probe the electronic structure of **1**, we employed SA-CASSCF-SO calculations in OpenMolcas 22.06 (see Experimental Section and the Supporting Information for details).^26^ The total crystal field splitting of the ^4^*I*_15/2_ ground multiplet is 179 cm^–1^ (Table S4), which is very small compared to bis-Cp^R^ Er^III^ complexes.^43^ Indeed, the first excited doublet is predicted to lie at 8 cm^–1^, which is approaching the limit of accuracy of the SA-CASSCF-SO methodology. This small splitting also results in notable mixing of the *m*_*J*_ functions as well as a strong rhombic character of the states. Nonetheless, the lowest three doublets have eas*y*-axis type magnetic anisotropy, with the easy axis pointing roughly along the Ln–Cp* centroids for both centers (Figure 4), suggesting that the Bi_6_^6–^ cluster seems to dominate over the Cp* ligand. This is in agreement with previous findings on the related Tb^III^ and Dy^III^ analogues that showed ground state main magnetic anisotropy axes perpendicular to the Ln–Cp* centroid axes,^7^ following the oblate-spheroidal nature of the 4f-electron densities for their largest angular momentum doublets.^14^

An estimation of the order of magnitude of the magnetic exchange coupling can be obtained by modeling the magnetic susceptibility in PHI,^44^ using the Lines model which only considers the spin-dependent component of the exchange coupling, eq 1 (Figures S16 and S18).^45^ The first term is the crystal field potential for each ion (fixed from SA-CASSCF-SO calculations in the global molecular reference frame; Table S5), the second is the Zeeman term, and the third is the spin–spin exchange between the two Er^III^ ions (implemented in the *J*, *m*_*J*_ basis via a Clebsch–Gordan transformation).
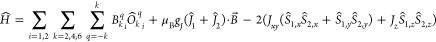
1

The magnetic susceptibility predicted by SA-CASSCF-SO calculations (i.e., a simulation using *J* = 0 and *g*_*J*_ = ^6^/_5_) slightly underpredicts the high temperature value though also fails to reproduce the low temperature profile, indicating the presence of nonzero exchange interactions (Figure S18). A fit of the data assuming isotropic exchange coupling (i.e., *J* = *J*_*xy*_ = *J*_*z*_) provides a very good reproduction of the experimental χ_M_*T* for *g*_*J*_ = 1.22 and *J* = −1.0 cm^–1^ (Figure S18). The fitted *g*_*J*_ value being slightly higher than the expected Landé *g* factor of ^6^/_5_ is likely an artifact due to a small error in the sample mass, rather than being a real effect. However, this model does not reproduce the reduced magnetization data well, which is sensitive to the low-lying states. Introducing some anisotropy in the exchange coupling provides a very good fit of both data sets with *g*_*J*_ = 1.23(1), *J*_*xy*_ = −5.62(8) cm^–1^ and *J*_*z*_ = +0.72(3) cm^–1^ (Figure 3). Interestingly, the fitted exchange coupling is dominantly antiferromagnetic, compared to the ferromagnetic coupling found for the Tb and Dy analogues, which have *J*_*xy*_ = −0.07 cm^–1^ and *J*_*z*_ = +2.77 cm^–1^ (Tb), and *J* ∼ 1 cm^–1^ (Dy).^7^ For all Tb, Dy, and Er analogues, the sign of the exchange coupling is opposite to that predicted on the basis of purely dipolar interactions between the single ions: the ground state easy axes for Tb and Dy are perpendicular to the internuclear axis, while for Er they are parallel, which would suggest anti- and ferromagnetic dipolar coupling, respectively. Hence, there must be a substantial superexchange coupling mediated via the Bi_6_^6–^ Zintl ion. It is curious that the nature of the superexchange is opposite for the two sets of ions with different inherent single-ion anisotropies (Tb/Dy vs Er). This could be probed further by preparation and study of the Yb analogue, which should have similar inherent anisotropy to the present Er example.^14^ For complex **1**, the dominant antiferromagnetic interactions explain the lack of observable slow magnetization dynamics (i.e., lack of SMM behavior).

## Conclusion

In conclusion, an unprecedented dinuclear erbium bismuth complex, **1**, with a neutral heterometallocubane core including a Bi_6_^6–^ Zintl ion was isolated. Notably, the smaller ionic radius of Er^III^ leads to stronger structural distortions within the cube moiety relative to the larger congeners containing Tb^III^, Dy^III^, and Y^III^. *Ab initio* calculations reveal that the Bi_6_^6–^ unit imparts an equatorial crystal field on the Er^III^ ions concomitant with enabling substantial antiferromagnetic superexchange coupling through diffuse Bi valence orbitals. It would be illustrative to prepare and study the putative [COT_2_Er_2_Bi_6_]^4–^ anion where the COT^2–^ ligand is known to provide an equatorial crystal field for Er^III^ ions^16,36,42,46−48^ and thus work constructively to support easy-axis anisotropy with the Bi_6_^6–^ ion. These findings show that we are just scratching the surface of the electronic and magnetic coupling with Zintl ion fragments and may lead to new design principles for magnetic materials comprising bismuth bridges and 4f-spin carriers.

## Supporting Information Available

Detailed synthetic procedures, crystallographic refinement parameters, UV–vis spectra, and magnetic and computational data (PDF)
