# Supplementary material for: An Organometallic Erbium Bismuth Cluster Complex Comprising a Bi66– Zintl Ion
Source: Inorg Chem. 2024 Oct 18;63(43):20250–6. doi: 10.1021/acs.inorgchem.4c02636 (PMC11523234; doi:10.1021/acs.inorgchem.4c02636)
Supplement: Supplementary file 1 — ic4c02636_si_001.pdf [file ic4c02636_si_001.pdf]

Supporting Information

for

An Organometallic Erbium Bismuth Cluster Complex Comprising a Bi_6^{6-} Zintl Ion

Florian Benner,[a] Elizabeth R. Pugliese,[a] Reece Q. Marsden,[b] Richard J. Staples,[a] Nicholas F. Chilton,*[b,c] Selvan Demir*[a]

[a] Department of Chemistry, Michigan State University, 578 South Shaw Lane, East Lansing, Michigan 48824, USA
[b] Department of Chemistry, The University of Manchester, Manchester M13 9PL, UK
[c] Research School of Chemistry, The Australian National University, Sullivans Creek Road, Canberra, ACT, 2601, Australia.

*Correspondence to:

nicholas.chilton@anu.edu.au (N.F.C.)

sdemir@chemistry.msu.edu (S.D.)

Inorg. Chem.

Table of Contents

1. Single Crystal X-Ray Diffraction S3

Table S1. Crystal data and structural refinement of $[K(THF)_4]_2[Cp^*{}_2Er_2Bi_6]$ (**1**) and $Cp^*{}_2ErPh(THF)$(**2**). **S3**

Table S2. Parameters for the powder X-ray diffraction data collection from the decomposition of $[K(THF)_4]_2[Cp^*{}_2Er_2Bi_6]$ (**1**). **S3**

Figure S1. Structure of $[K(THF)_4]_2[Cp^*{}_2Er_2Bi_6]$ (**1**). **S4**

Figure S2. Space filling model of $[Cp^*{}_2Er_2Bi_6]$ in a crystal of **1**. **S5**

Figure S3. Structure of $Cp^*{}_2ErPh(THF)$. **S6**

Table S3. Metrical parameters for the Bi–Bi–Bi angles (°) of the $Bi_6{}^{6-}$ moiety in $[K(THF)_4]_2[Cp^*{}_2RE_2Bi_6]$. **S7**

Figure S4. Superposition of an idealized cube (yellow) with $\{Er_2Bi_6\}$ wireframe model in top and side view. **S8**

Figure S5. Select metrical parameters for the smallest and largest angles within the $\{Er_2Bi_6\}$ cubane moiety of **1**. **S8**

Figure S6. Powder X-ray diffraction pattern of Bi^0 formed during the decomposition of **1** (black) and reported pattern of Bi^0 (red). **S9**

2. UV-Vis Spectroscopy S10

Figure S7. UV-Vis spectra of $[K(THF)_4]_2[Cp^*{}_2Er_2Bi_6]$ (**1**) recorded at 50 µmol/L at room temperature over the course of ten scans (absorbance). **S10**

Figure S8. UV-Vis spectra of $[K(THF)_4]_2[Cp^*{}_2Er_2Bi_6]$ (**1**) recorded at 50 µmol/L at room temperature (scan 1, absorbance). **S11**

Figure S9. UV-Vis spectra of $[K(THF)_4]_2[Cp^*{}_2Er_2Bi_6]$ (**1**) recorded at 50 µmol/L at room temperature over the course of ten scans (ε). **S12**

Figure S10. UV-Vis spectrum of $[K(THF)_4]_2[Cp^*{}_2Er_2Bi_6]$ (**1**) recorded at 50 µmol/L at room temperature (scan 1, ε). **S13**

3. IR Spectroscopy S14

Figure S11. FTIR spectrum of $[K(THF)_4]_2[Cp^*{}_2Er_2Bi_6]$ (**1**). **S14**

4. Magnetic Measurements S15

Figure S12. Variable-temperature dc susceptibility data of polycrystalline $[K(THF)_4]_2[Cp^*{}_2Er_2Bi_6]$ (**1**), collected under a 0.5 T applied dc field. **S15**

Figure S13. Variable-temperature dc susceptibility data of polycrystalline $[K(THF)_4]_2[Cp^*{}_2Er_2Bi_6]$ (**1**). **S16**

Figure S14. Isothermal variable-field magnetization (*M*) data collected for $[K(THF)_4]_2[Cp^*{}_2Er_2Bi_6]$ (**1**). **S17**

Figure S15. Isothermal variable-field magnetization (*M*) data collected for [K(THF)$_4$]$_2$[Cp*$_2$Er$_2$Bi$_6$] (**1**) collected at 1.8 K. **S18**

5. *Ab initio* Calculations **S19**

Table S4. SA-CASSCF-SO-calculated crystal field splitting of the *J* = 15/2 multiplet of **1**. **S19**

Table S5. SA-CASSCF-SO-calculated crystal field parameters. **S19**

Figure S16. Variable-temperature dc susceptibility data of polycrystalline [K(THF)$_4$]$_2$[Cp*$_2$Er$_2$Bi$_6$] (**1**), collected under 0.5 T dc field with predicted susceptibility from CASSCF-SO calculations. **S21**

Figure S17. Variable temperature field-dependent magnetization curves recorded [K(THF)$_4$]$_2$[Cp*$_2$Er$_2$Bi$_6$] (**1**) between 0 and 7 T at 2, 4, 6, 8, and 10 K with predicted magnetization from CASSCF-SO calculations. **S22**

Figure S18. Variable-temperature DC susceptibility data of **1** with predicted susceptibility for *J* = 0 from CASSCF-SO calculations and fitted data. **S23**

6. References **S23**

1. Single Crystal X-Ray Diffraction

Table S1. Crystal data and structural refinement of $[K(THF)_4]_2[Cp*_2Er_2Bi_6]$ (**1**) and $Cp*_2ErPh(THF)$ (**2**).

Compound	**1**	**2**
Empirical formula	$C_{52}H_{94}Bi_6K_2O_8Er_2$	$C_{30}H_{43}OEr$
CCDC number	2353572	2353575
Formula weight (g mol^{-1})	2513.87	586.90
Temperature (K)	100.0(1)	100.0(1)
Crystal system	Monoclinic	Monoclinic
Space group	$P2_1/n$	$P2_1$/c
Unit Cell Dimensions	a = 15.2839(3) Å	a = 9.4402(2) Å
	b = 16.7910(3) Å	b = 17.1224(3) Å
	c = 40.2427(7) Å	c = 16.7534(3) Å
	α = 90°	α = 90°
	β = 100.957(2)°	β = 102.689(2)°
	γ = 90°	γ = 90°
Volume (Å^3)	10139.3(3)	2641.86(9)
Z	6	4
ρ_{calc} (g cm^{-3})	2.470	1.476
μ (mm^{-1})	18.180	3.195
F(000)	6852.0	1196.0
Crystal size (mm^3)	0.278 x 0.175 x 0.112	0.3 x 0.257 x 0.106
Radiation	Mo K_α (λ = 0.71073)	Mo K_α (λ = 0.71073)
2Θ range for data collection (°)	4.96 to 63.776	5.022 to 62.122
Reflections collected	144304	49733
Independent reflections	29761 R_{int} = 0.0887	7328 R_{int} = 0.0432
Data/restraints/parameters	29761/55/633	7328/0/299
Goodness-of-fit on F^2	1.180	1.054
Final R indexes [I>=2σ (I)]	R_1 = 0.0796, wR_2 = 0.1336	R_1 = 0.0276, wR_2 = 0.0538
Final R indexes [all data]	R_1 = 0.1305, wR_2 = 0.1441	R_1 = 0.0392, wR_2 = 0.0575
Largest diff. peak/hole (e Å^{-3})	2.15/-1.85	1.22/-0.84

Table S2. Parameters for the powder X-ray diffraction data collection from the decomposition of $[K(THF)_4]_2[Cp*_2Er_2Bi_6]$ (**1**).

Detector	Linxeye
Anode voltage	40 kV
Anode current	40 mA
Primary Optics	Global Mirror, 1.5 mm slit, 2.5°soller
Secondary Optics	Dectector Slit 3.0mm 2.5°soller
Sampling steps	0.02 °/ step, 0.1 sec /step
Goniometer	theta 2-theta
Scan Type	Coupled Theta/2theta
Detector	2.8° opening

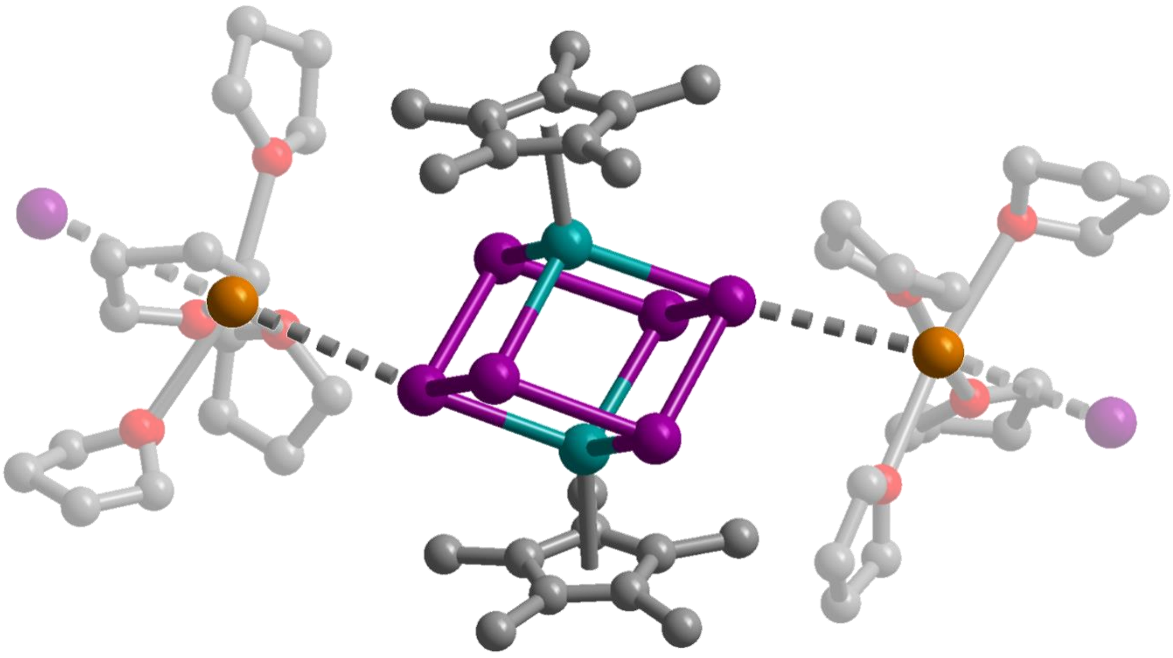

Figure S1. Structure of $[K(THF)_4]_2[Cp^*{}_2Er_2Bi_6]$ (**1**). Teal, purple, orange, red, and gray spheres represent Er, Bi, K, O, and C atoms, respectively. The THF molecules have been faded for clarity.

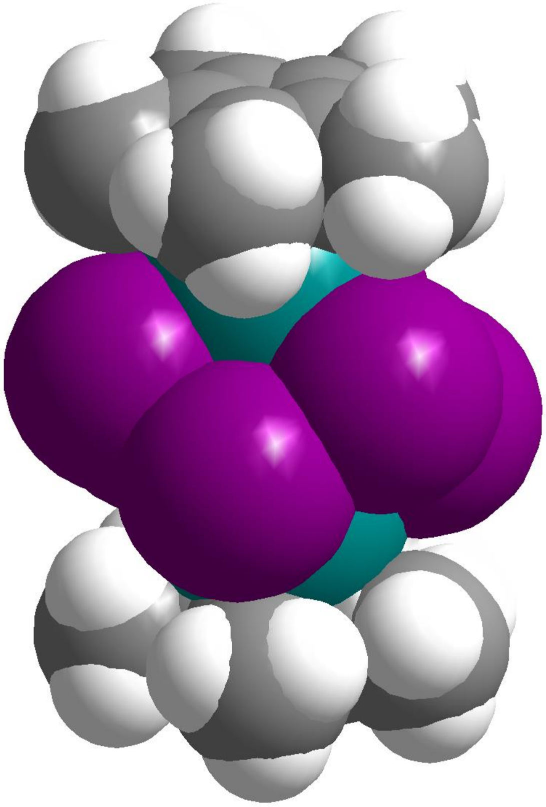

Figure S2. Space filling model of $[Cp^*_2Er_2Bi_6]^{2-}$ in a crystal of **1**. Teal, purple, gray, and white spheres represent Er, Bi, C, and H atoms, respectively. The $[K(THF)_4]^+$ cations have been omitted for clarity.

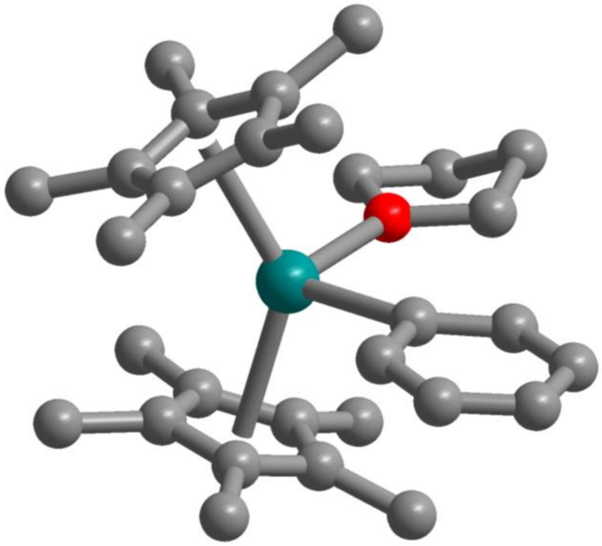

Figure S3. Structure of Cp*$_2$ErPh(THF) **2**. Teal, red, and gray spheres represent Er, O, and C and atoms, respectively. The H atoms have been omitted for clarity.

Table S3. Metrical parameters for the Bi–Bi–Bi angles (°) of the Bi_6^{6-} moiety in $[K(THF)_4]_2[Cp^*{}_2RE_2Bi_6]$ (where RE = Tb, Dy, Y, Er), with corresponding angle labels.

	Angles (°)			
	**Tb**	**Dy**	**Y**	**Er**
**1**	102.4	102.3	102.0	100.8
**2**	102.7	102.1	102.6	101.7
**3**	103.4	103.1	103.6	100.8
**4**	102.4	102.3	102.0	103.0
**5**	102.7	102.1	102.6	103.4
**6**	103.4	103.1	103.6	101.5

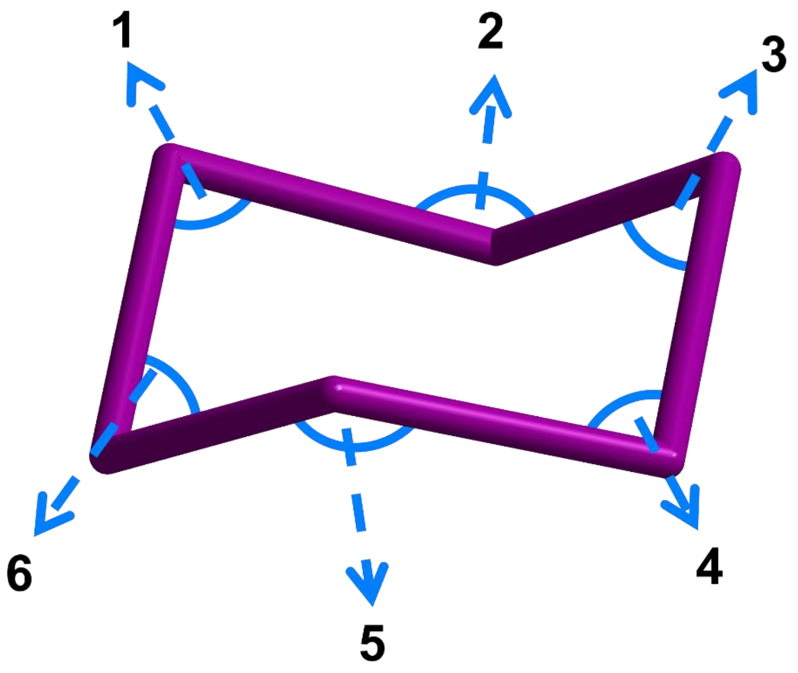

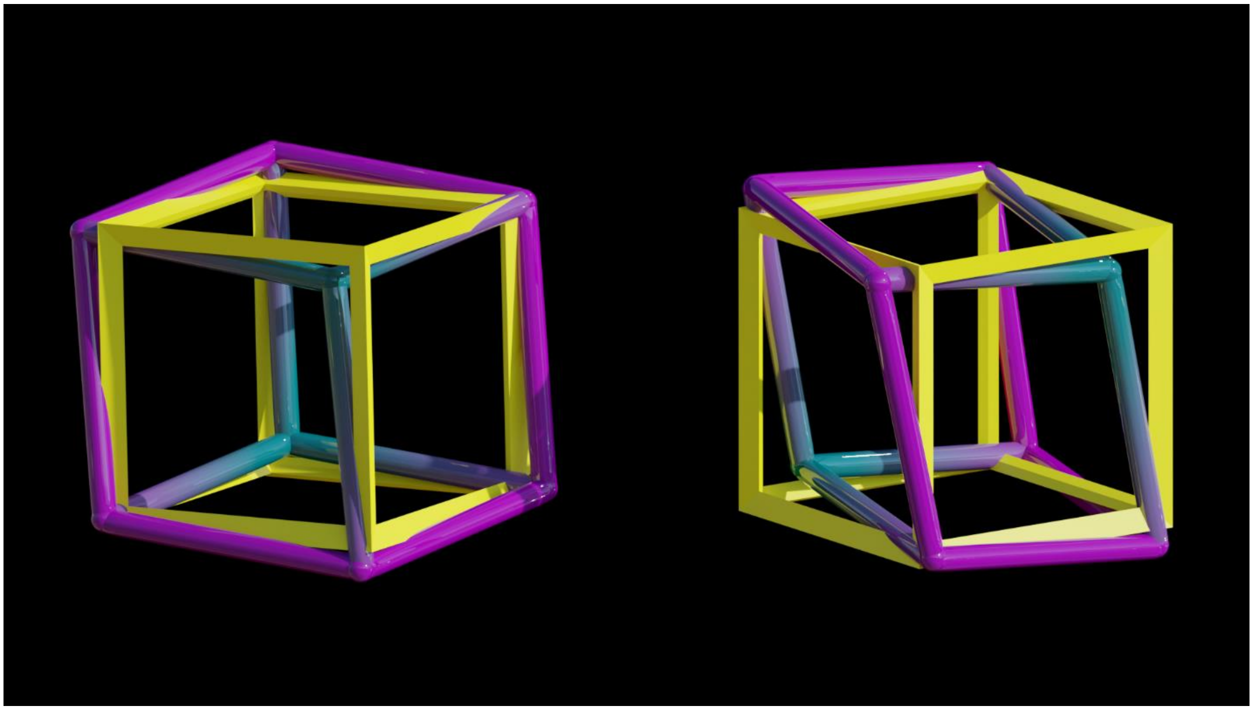

Figure S4. Superposition of an idealized cube (yellow) with {Er_2Bi_6} wireframe model in top and side view, highlighting structural distortions in **1**.

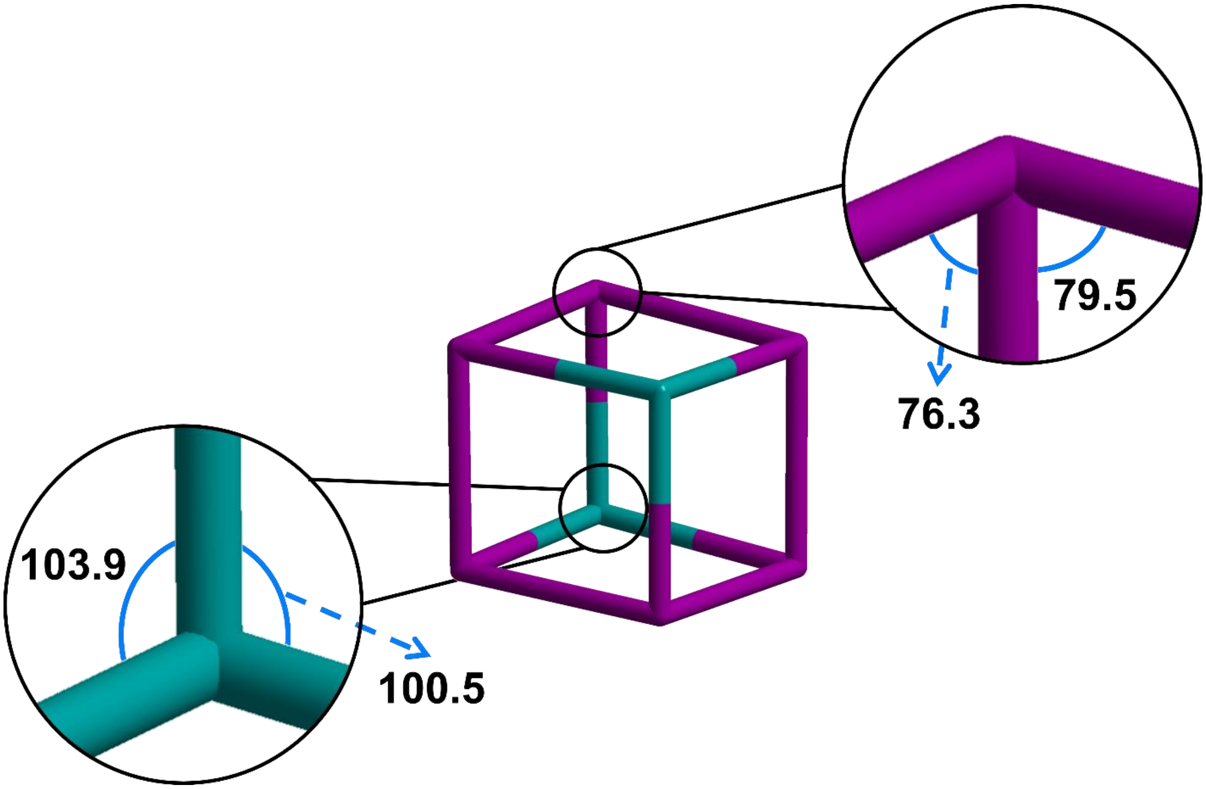

Figure S5. Select metrical parameters for the smallest and largest angles (°) within the {Er_2Bi_6} cubane moiety of **1**.

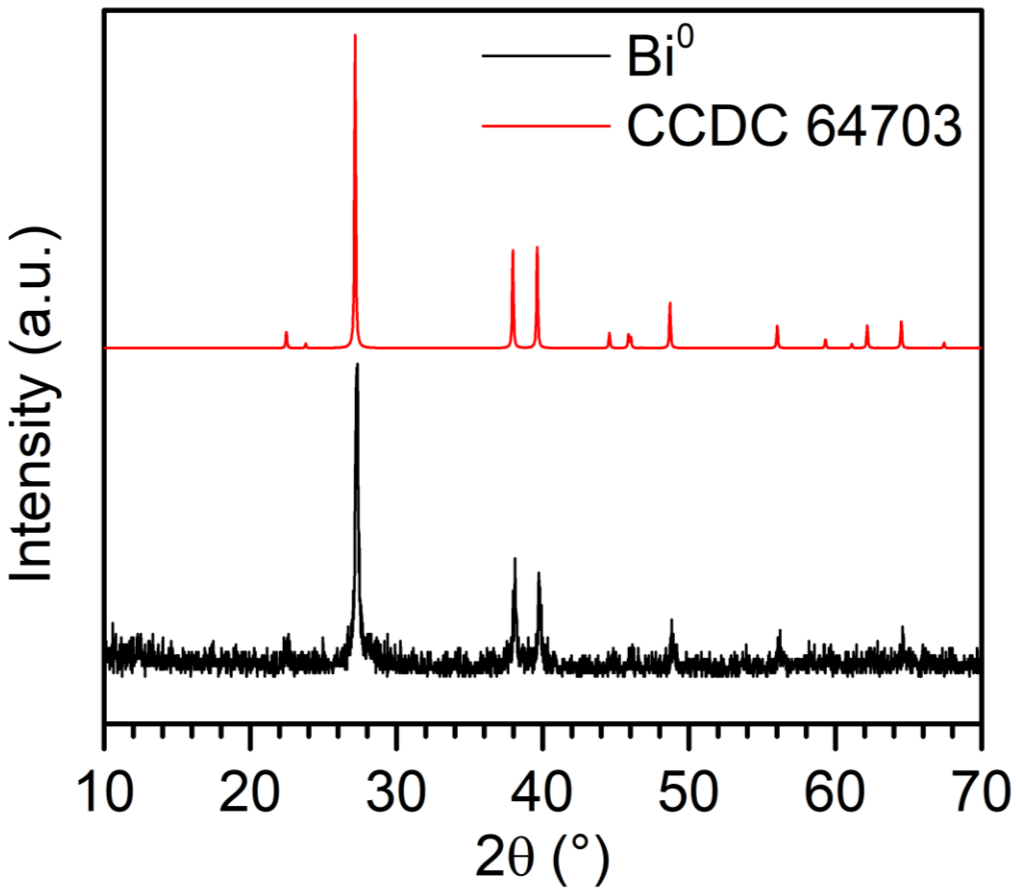

Figure S6. Powder X-ray diffraction pattern of Bi^0 formed during the decomposition of **1** (black) and reported pattern of Bi^0 (red).[1]

2. UV-Vis Spectroscopy

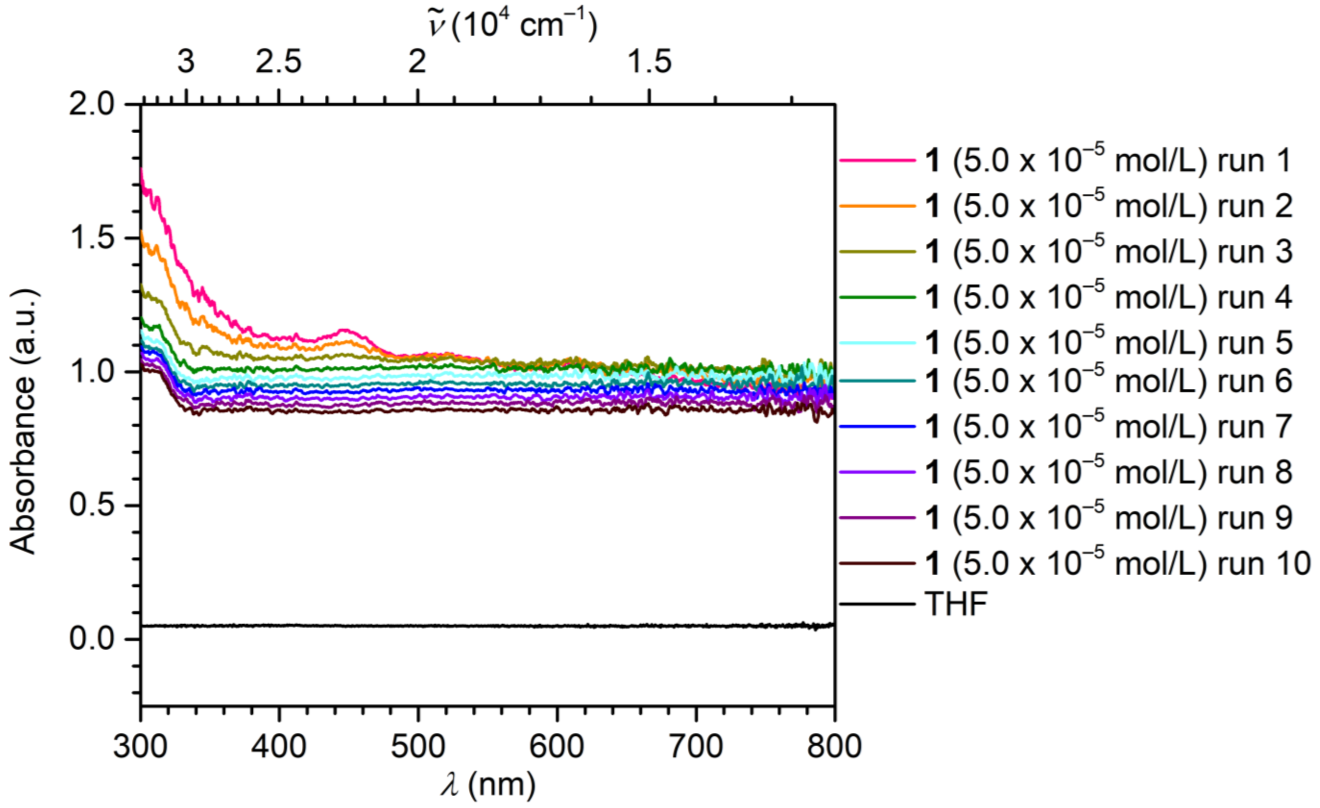

Figure S7. UV-Vis spectra of $[K(THF)_4]_2[Cp^*{}_2Er_2Bi_6]$ (**1**) recorded at 50 µmol/L at room temperature over the course of ten scans. A smoothing method was applied to reduce noise from data. Adjacent averaging with points of window set to 5 was implemented via Origin.

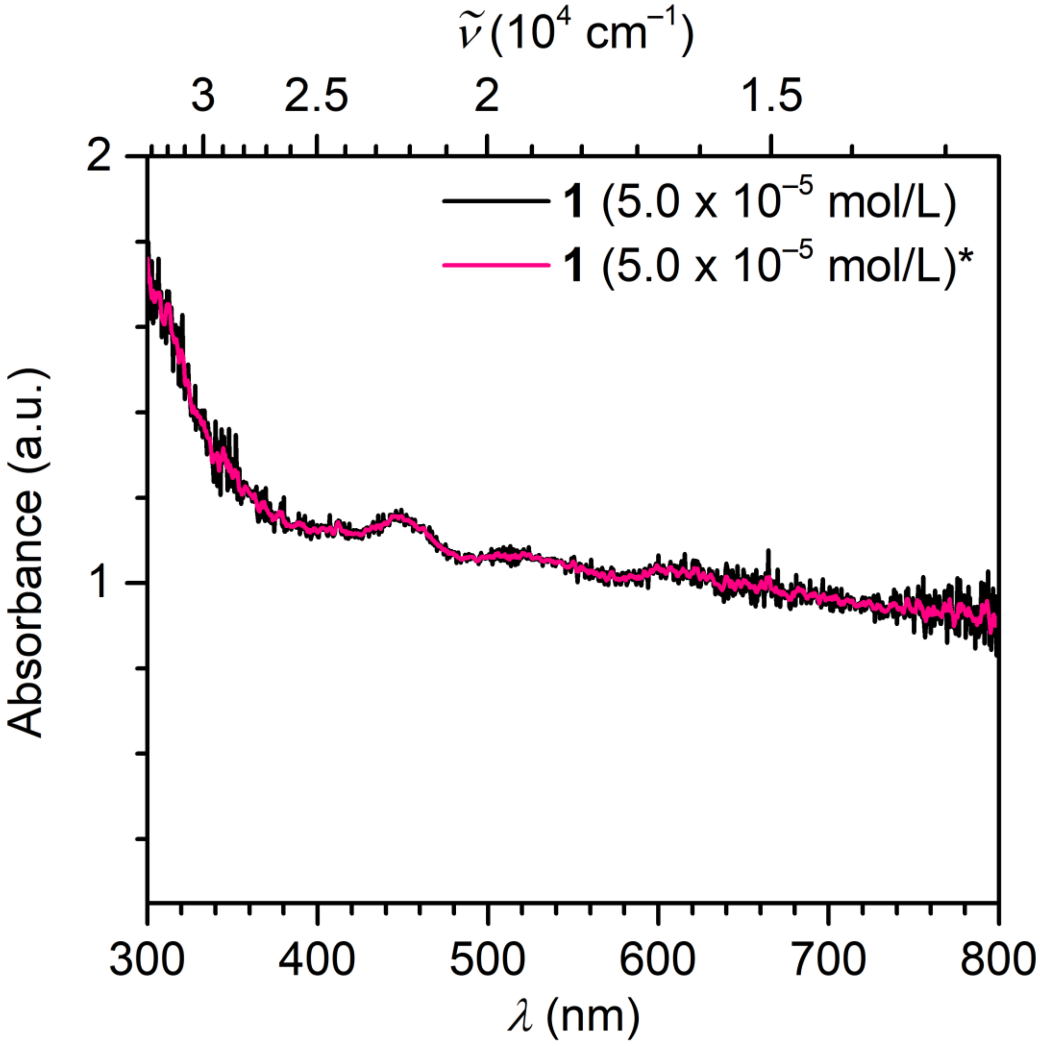

Figure S8. UV-Vis spectra of $[K(THF)_4]_2[Cp^*{}_2Er_2Bi_6]$ (**1**) recorded at 50 μmol/L at room temperature (scan 1). *Denotes a smoothing method was applied to reduce noise from data. Adjacent averaging with points of window set to 5 was implemented via Origin.

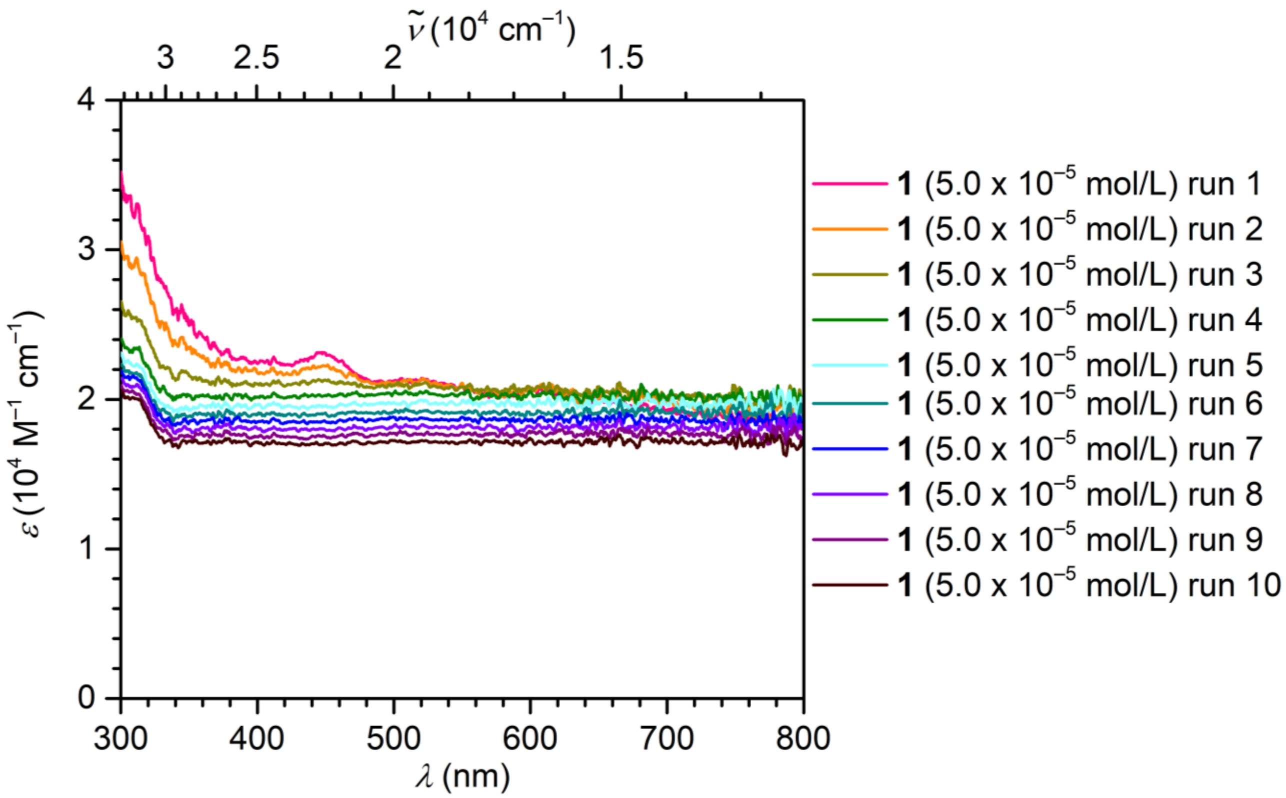

Figure S9. UV-Vis spectra of $[K(THF)_4]_2[Cp^*{}_2Er_2Bi_6]$ (**1**) recorded at 50 µmol/L at room temperature over the course of ten scans. A smoothing method was applied to reduce noise from data. Adjacent averaging with points of window set to 5 was implemented via Origin.

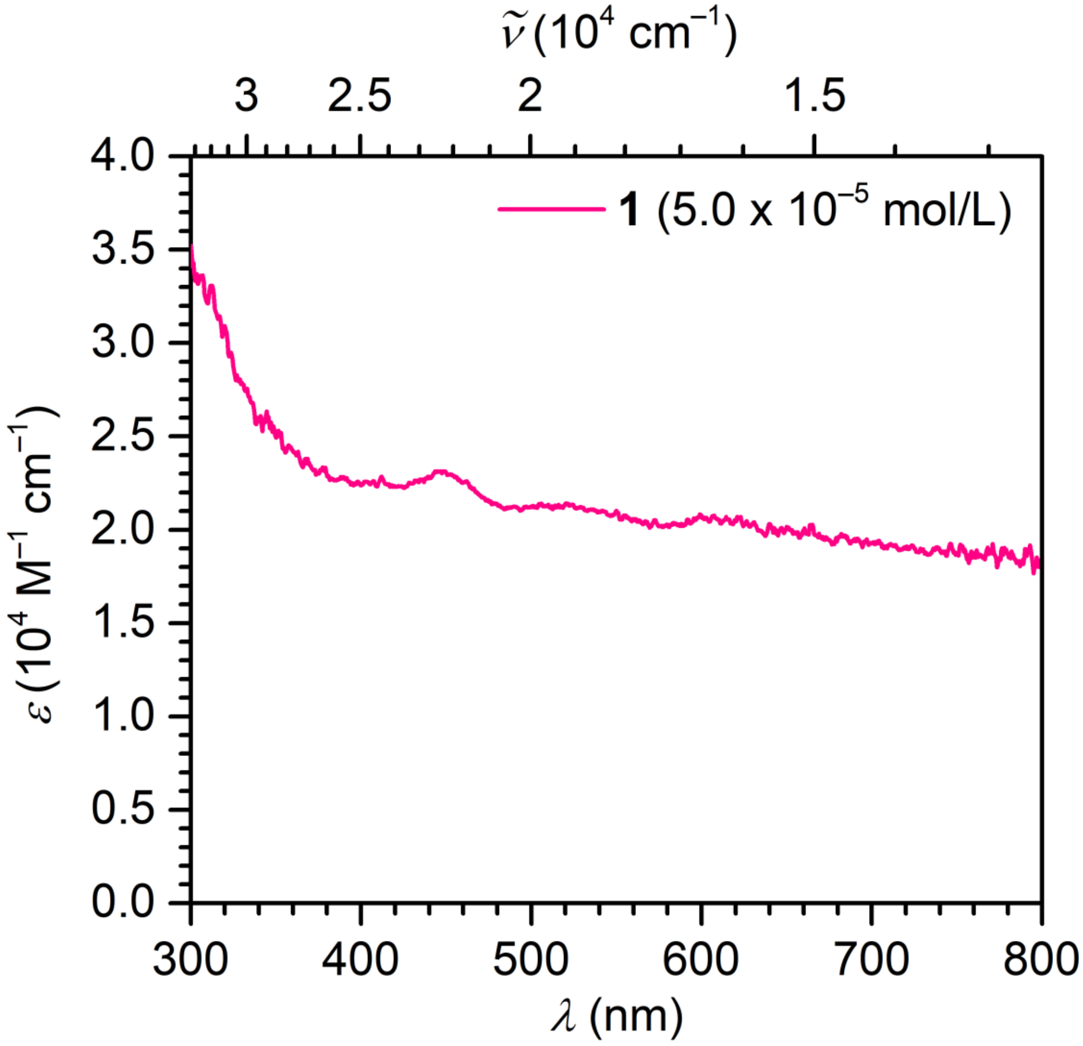

Figure S10. UV-Vis spectrum of $[K(THF)_4]_2[Cp^*{}_2Er_2Bi_6]$ (**1**) recorded at 50 µmol/L at room temperature (scan 1). A smoothing method was applied to reduce noise from data. Adjacent averaging with points of window set to 5 was implemented via Origin.

3. IR Spectroscopy

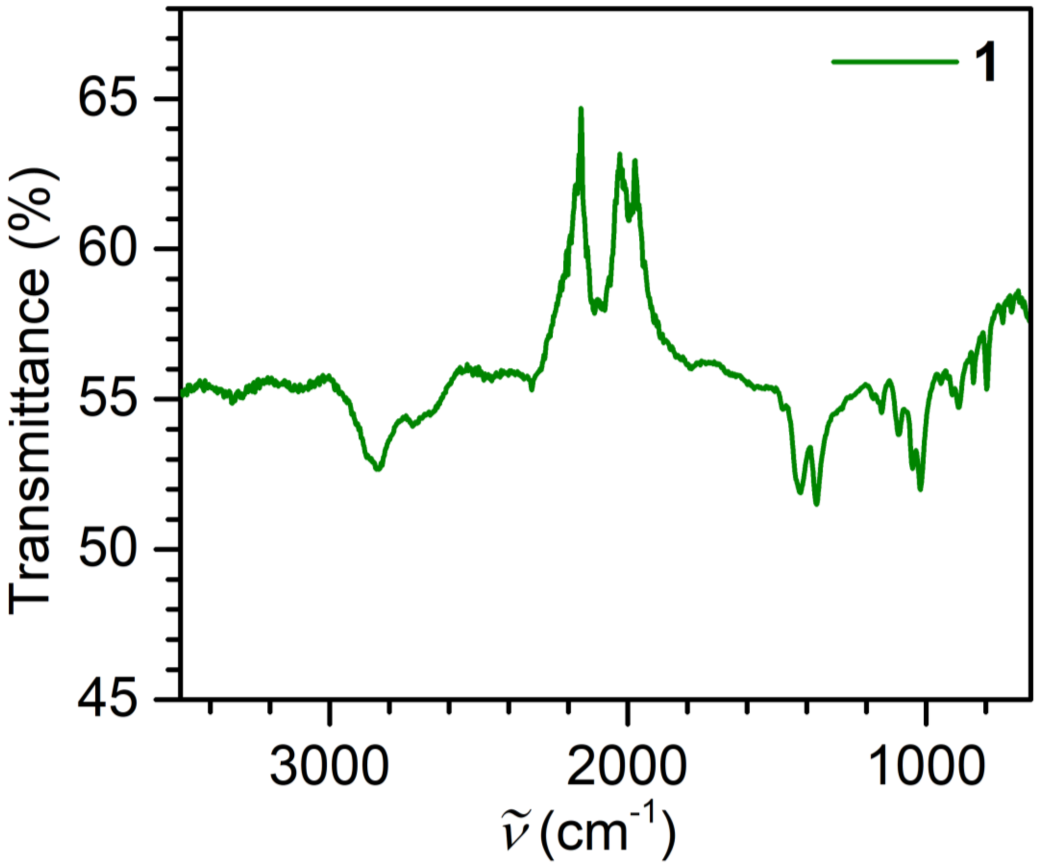

Figure S11. FTIR spectrum of $[K(THF)_4]_2[Cp^*{}_2Er_2Bi_6]$ (**1**) measured on crushed crystalline solids under an inert nitrogen atmosphere.

4. Magnetic Measurements

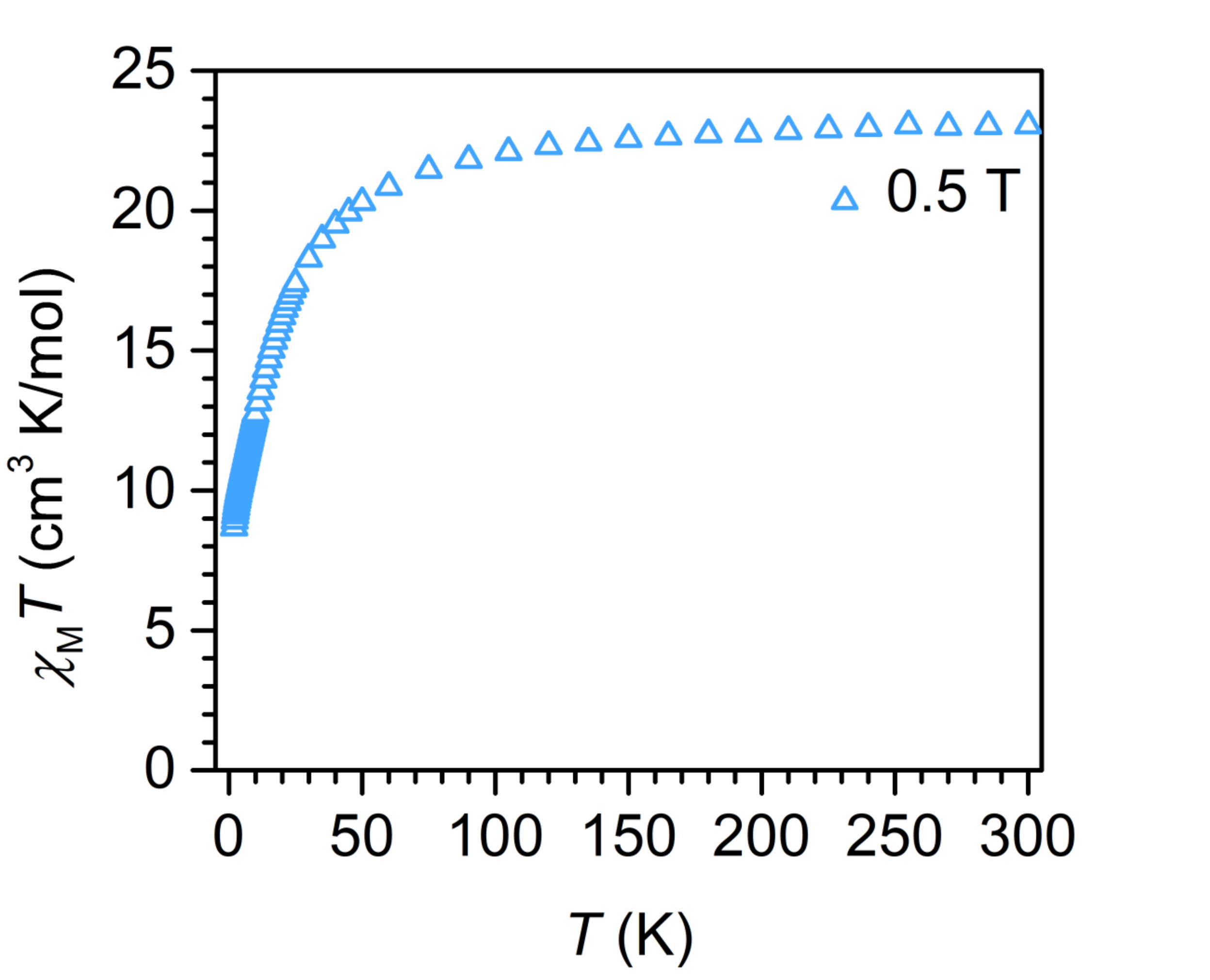

Figure S12. Variable-temperature dc susceptibility data of polycrystalline $[K(THF)_4]_2[Cp^*_2Er_2Bi_6]$ (**1**), collected under a 0.5 T (blue triangles) applied dc field.

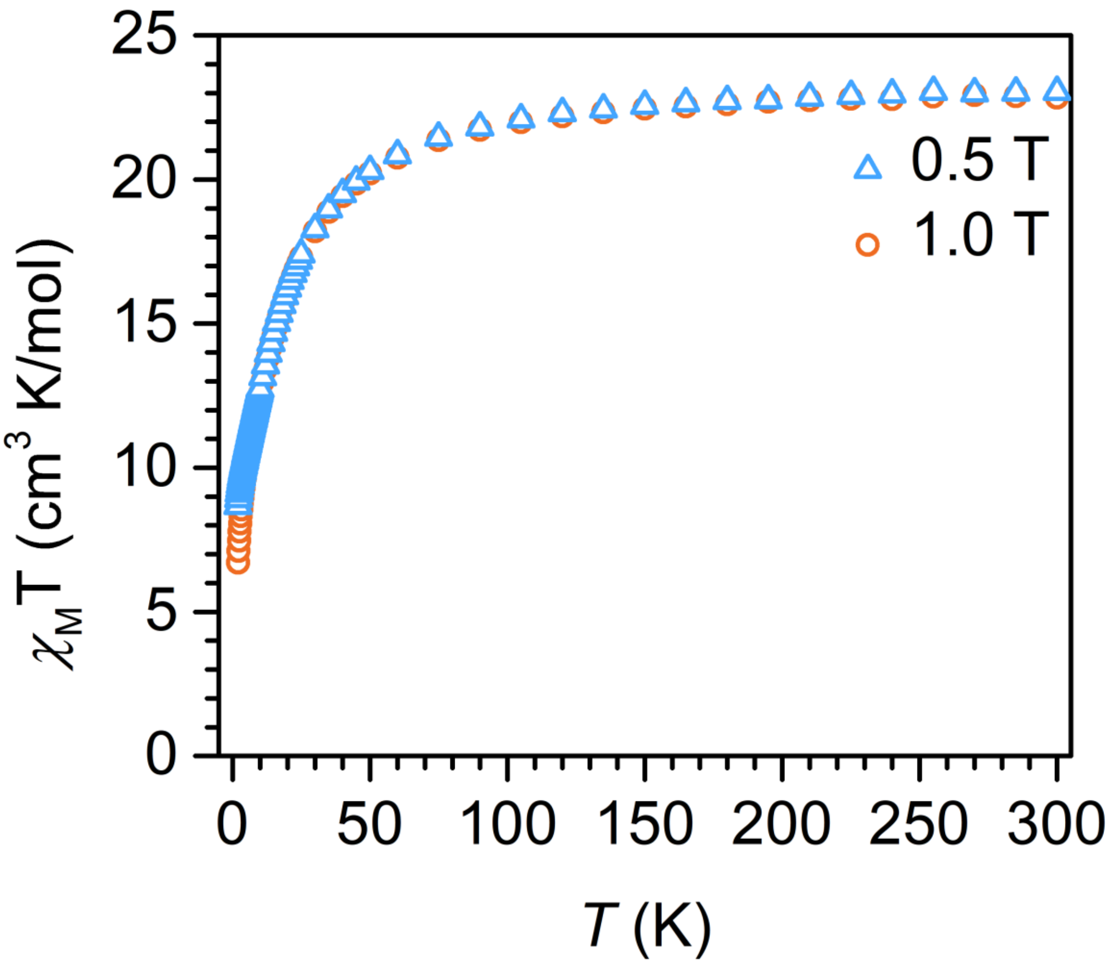

Figure S13. Variable-temperature dc susceptibility data of polycrystalline $[K(THF)_4]_2[Cp^*{}_2Er_2Bi_6]$ (**1**), collected under 0.5 T (blue triangles) and 1.0 T (orange circles) applied dc fields.

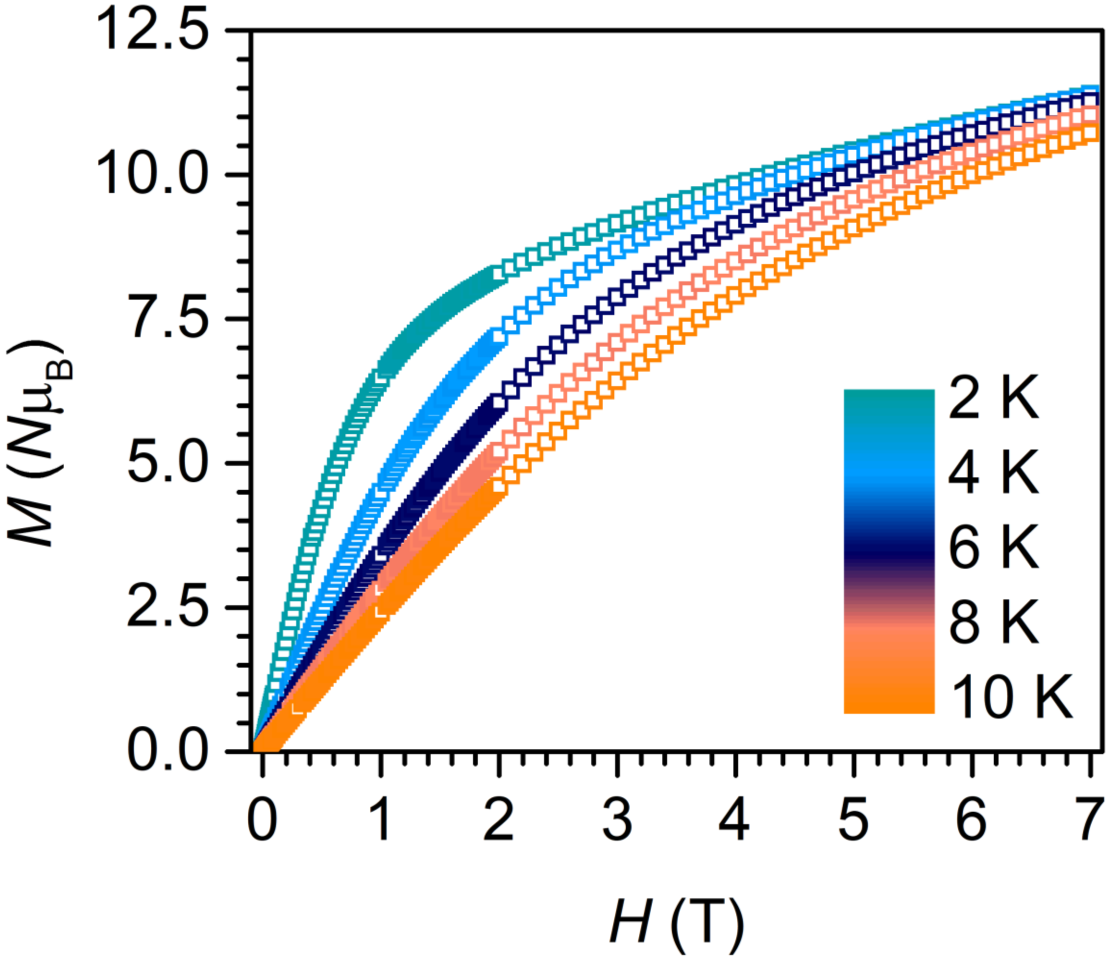

Figure S14. Variable temperature field-dependent magnetization curves recorded for $[K(THF)_4]_2[Cp^*{}_2Er_2Bi_6]$ (**1**). Measurements were carried out between 0 and 7 T at 2, 4, 6, 8, and 10 K.

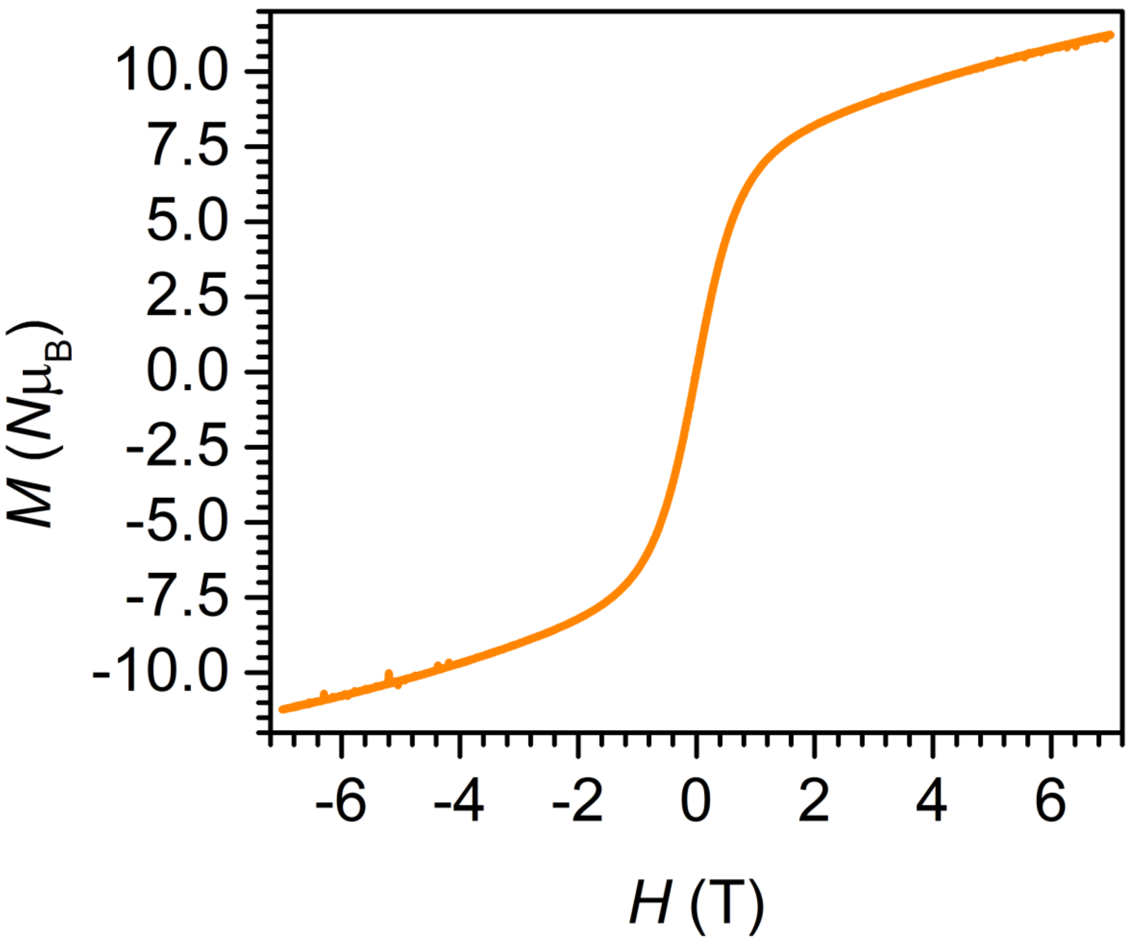

Figure S15. Isothermal variable-field magnetization (*M*) data collected for $[K(THF)_4]_2[Cp^*{}_2Er_2Bi_6]$ (**1**) collected at 1.8 K with an average sweep rate of 0.01 T s^{-1}.

5. *Ab initio* Calculations

Table S4. SA-CASSCF-SO-calculated crystal field splitting of the J = 15/2 multiplet of **1**. Wavefunctions given to nearest 1% with contributions > 5% shown.

Energy (cm^{-1})	g_1	g_2	g_3	Wavefunction
0	14.2	1.04	0.35	$49\% \left\|\mp\frac{15}{2}\right\rangle + 31\% \left\|\mp\frac{9}{2}\right\rangle + 13\% \left\|\mp\frac{7}{2}\right\rangle$
8	10.1	4.11	3.26	$63\% \left\|\mp\frac{7}{2}\right\rangle + 20\% \left\|\mp\frac{15}{2}\right\rangle + 7\% \left\|\mp\frac{5}{2}\right\rangle$
36	12.6	0.67	0.54	$57\% \left\|\mp\frac{9}{2}\right\rangle + 27\% \left\|\mp\frac{15}{2}\right\rangle + 5\% \left\|\mp\frac{11}{2}\right\rangle$
59	5.33	5.01	4.30	$46\% \left\|\pm\frac{5}{2}\right\rangle + 33\% \left\|\mp\frac{5}{2}\right\rangle + 13\% \left\|\mp\frac{7}{2}\right\rangle$
107	12.36	0.22	0.05	$80\% \left\|\pm\frac{11}{2}\right\rangle + 7\% \left\|\pm\frac{9}{2}\right\rangle + 7\% \left\|\pm\frac{13}{2}\right\rangle$
121	3.42	1.19	0.94	$79\% \left\|\mp\frac{3}{2}\right\rangle + 7\% \left\|\mp\frac{1}{2}\right\rangle$
165	8.01	5.75	3.29	$57\% \left\|\mp\frac{1}{2}\right\rangle + 16\% \left\|\mp\frac{13}{2}\right\rangle + 12\% \left\|\pm\frac{13}{2}\right\rangle + 5\% \left\|\mp\frac{3}{2}\right\rangle$
179	13.10	2.32	1.87	$61\% \left\|\pm\frac{13}{2}\right\rangle + 20\% \left\|\pm\frac{1}{2}\right\rangle + 7\% \left\|\mp\frac{1}{2}\right\rangle$

Table S5. SA-CASSCF-SO-calculated crystal field parameters.

Parameter	Er1 (cm^{-1})	Er2 (cm^{-1})
B_2^{-2}	1.337798E-01	-1.052902E-01
B_2^{-1}	2.161027E-01	5.147214E-01
B_2^{0}	-2.059888E-01	-1.122087E-01
B_2^{1}	-1.628709E-01	2.086725E-01
B_2^{2}	1.645812E-02	3.827166E-02
B_4^{-4}	-6.950512E-06	-4.699817E-04
B_4^{-3}	-1.245523E-03	2.921138E-03
B_4^{-2}	-1.904467E-04	-1.363257E-04

B_4^{-1}	9.738368E-04	-1.112262E-03
B_4^0	2.637409E-04	1.857009E-04
B_4^1	-6.676792E-04	1.835828E-04
B_4^2	2.961699E-04	-5.137075E-04
B_4^3	1.967231E-03	2.250927E-03
B_4^4	2.638848E-05	6.903153E-04
B_6^{-6}	-1.149776E-04	9.813915E-05
B_6^{-5}	-6.783260E-06	-5.766255E-05
B_6^{-4}	5.189588E-06	-8.135460E-05
B_6^{-3}	-8.796657E-05	-6.499669E-05
B_6^{-2}	1.545520E-05	-1.022152E-05
B_6^{-1}	-1.512509E-04	4.620533E-04
B_6^0	-5.942637E-05	-2.023907E-05
B_6^1	1.209669E-04	-7.347283E-05
B_6^2	1.537134E-05	2.380047E-04
B_6^3	1.411451E-04	6.525141E-05
B_6^4	-6.190240E-07	4.145080E-05
B_6^5	-1.227076E-04	1.572432E-04
B_6^6	5.673178E-05	2.211940E-05

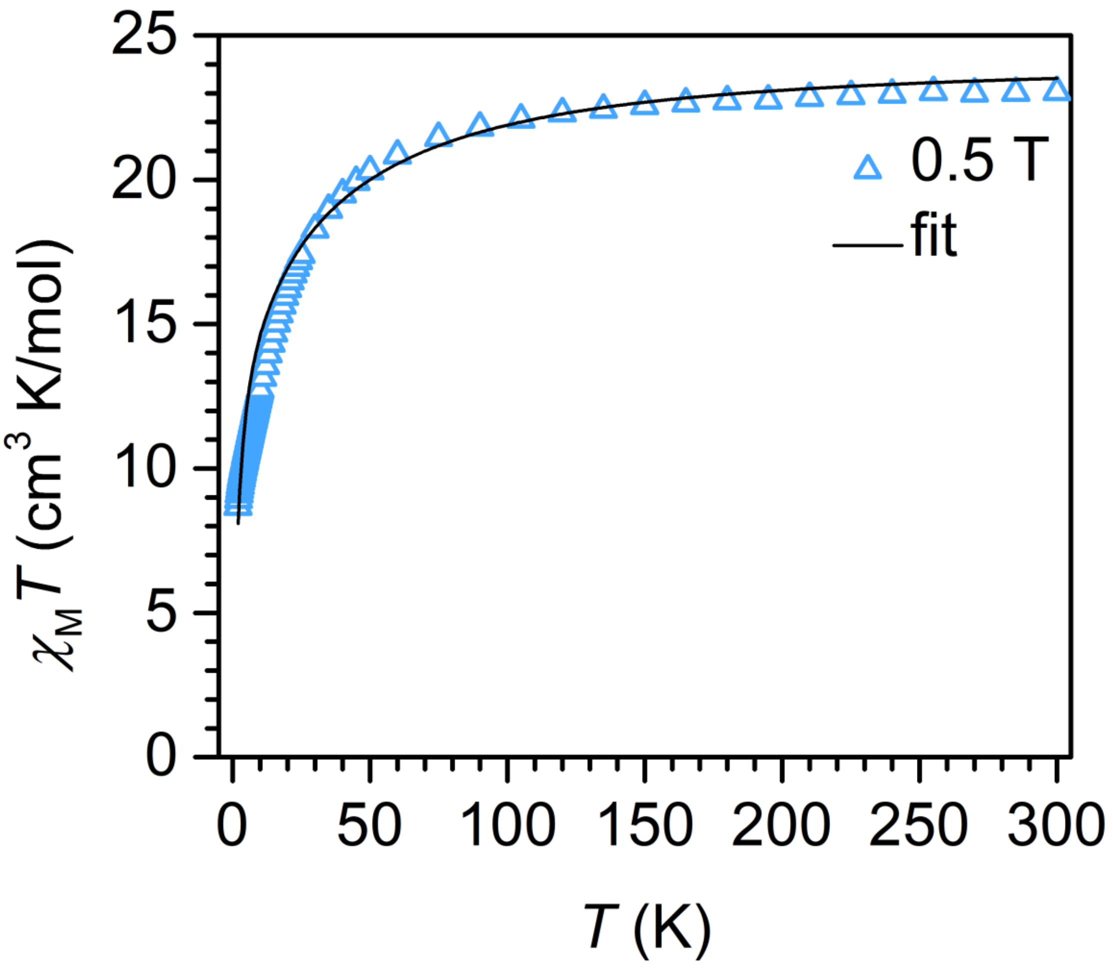

Figure S16. Variable-temperature dc susceptibility data of polycrystalline $[K(THF)_4]_2[Cp^*{}_2Er_2Bi_6]$ (**1**), collected under 0.5 T (blue triangles) dc field with predicted susceptibility from CASSCF-SO calculations.

.

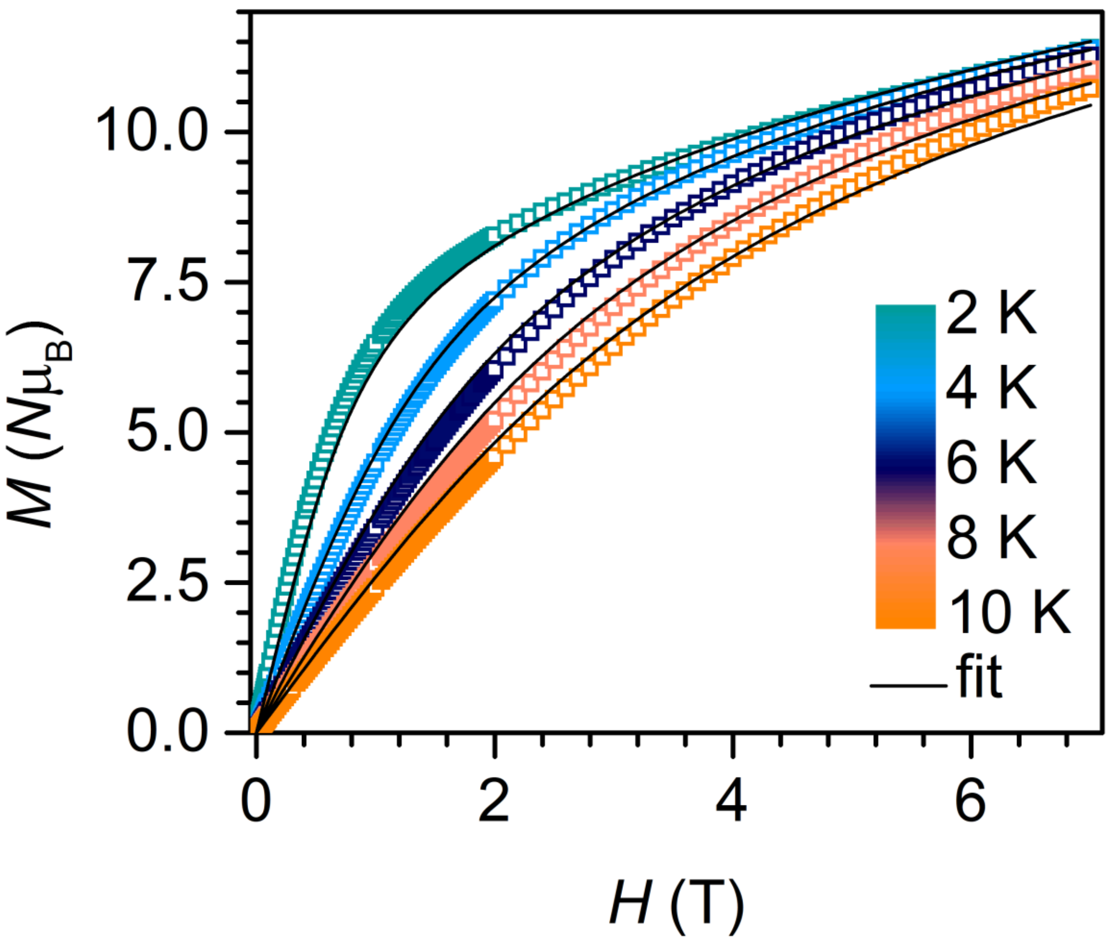

Figure S17. Variable temperature field-dependent magnetization curves recorded $[K(THF)_4]_2[Cp^*{}_2Er_2Bi_6]$ (**1**) between 0 and 7 T at 2, 4, 6, 8, and 10 K with predicted magnetization from CASSCF-SO calculations.

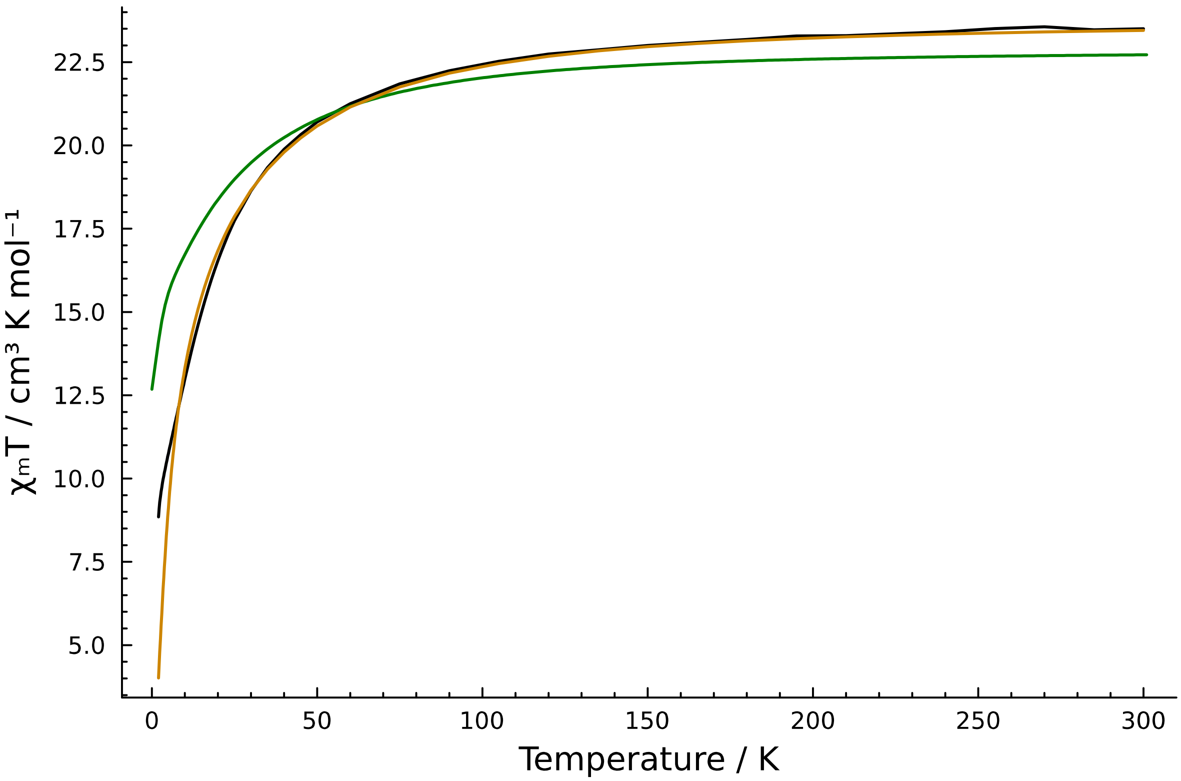

Figure S18. Variable-temperature DC susceptibility data of **1** collected under a 0.5 T applied magnetic field (black). Predicted susceptibility for $J = 0$ from CASSCF-SO calculations (green). Fitted data using PHI ($g_J = 1.22$ and $J = -1.0\ \mathrm{cm}^{-1}$, orange).

6. References

(1) Cucka, P.; Barrett, C. S. The Crystal Structure of Bi and of Solid Solutions of Pb, Sn, Sb and Te in Bi. *Acta Crystallogr.* **1962**, *15* (9). https://doi.org/10.1107/S0365110X62002297.
